# The role of inflammasomes in human diseases and their potential as therapeutic targets

**DOI:** 10.1038/s41392-023-01687-y

**Published:** 2024-01-05

**Authors:** Jing Yao, Keenan Sterling, Zhe Wang, Yun Zhang, Weihong Song

**Affiliations:** 1 The National Clinical Research Center for Geriatric Disease, Xuanwu Hospital, Capital Medical University, Beijing, 100053 China; 2 Townsend Family Laboratories, Department of Psychiatry, Brain Research Center, The University of British Columbia, 2255 Wesbrook Mall, Vancouver, BC V6T 1Z3 Canada; 3 Key Laboratory of Neurodegenerative Diseases, Ministry of Education, Beijing, P.R. China; 4 Zhejiang Clinical Research Center for Mental Disorders, Key Laboratory of Alzheimer’s Disease of Zhejiang Province, School of Mental Health and The Affiliated Kangning Hospital, Institute of Aging, Wenzhou Medical University, Wenzhou, Zhejiang 325000 China; 5 Oujiang Laboratory (Zhejiang Lab for Regenerative Medicine, Vision and Brain Health), Wenzhou, Zhejiang 325000 China

**Keywords:** Immunology, Medical research

## Abstract

Inflammasomes are large protein complexes that play a major role in sensing inflammatory signals and triggering the innate immune response. Each inflammasome complex has three major components: an upstream sensor molecule that is connected to a downstream effector protein such as caspase-1 through the adapter protein ASC. Inflammasome formation typically occurs in response to infectious agents or cellular damage. The active inflammasome then triggers caspase-1 activation, followed by the secretion of pro-inflammatory cytokines and pyroptotic cell death. Aberrant inflammasome activation and activity contribute to the development of diabetes, cancer, and several cardiovascular and neurodegenerative disorders. As a result, recent research has increasingly focused on investigating the mechanisms that regulate inflammasome assembly and activation, as well as the potential of targeting inflammasomes to treat various diseases. Multiple clinical trials are currently underway to evaluate the therapeutic potential of several distinct inflammasome-targeting therapies. Therefore, understanding how different inflammasomes contribute to disease pathology may have significant implications for developing novel therapeutic strategies. In this article, we provide a summary of the biological and pathological roles of inflammasomes in health and disease. We also highlight key evidence that suggests targeting inflammasomes could be a novel strategy for developing new disease-modifying therapies that may be effective in several conditions.

## Introduction

**Fig. 1:**
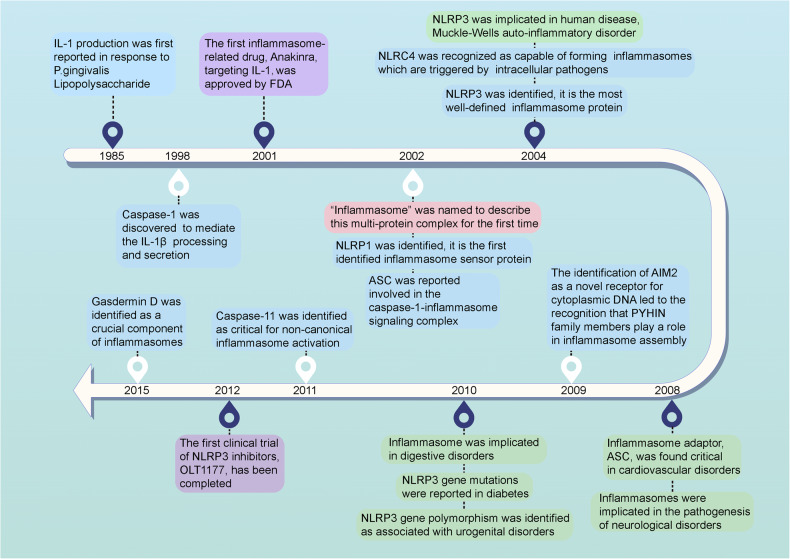
Milestone events in inflammasome-related research and their applications. Blue box: discoveries of key components of different inflammasomes, purple box: representative clinical applications of inflammasome-related modulators, green box: the association between inflammasomes and various human diseases, pink box: the origin of the term “inflammasome”. The figure was created with the assistance of FIGDRAW

The innate immune response enables humans to defend against new pathogens, environmental irritants, and tissue damage in part by triggering inflammation when immune cells recognize molecules that are commonly found in many pathogens or damaged cells but are otherwise absent in the body.^1^ This inflammatory response is mediated by large protein complexes called inflammasomes that have been increasingly shown to play a vital role in the immune system. The history of inflammasome-related research dates back to 1985, when Hanazawa and colleagues first showed that exposure to Lipopolysaccharide (LPS) induces interleukin-1 (IL-1) production in murine peritoneal macrophages (Fig. 1).^2^ Ultimately, this study was the first to suggest the existence of specific intracellular molecular platforms that could trigger the inflammatory response by inducing proinflammatory caspase activation and pro-IL-1β or pro-IL-18 processing.^3^ In the 1990s, caspase-1-mediated IL-1β processing and secretion was discovered and characterized, which provided the first tangible evidence that a molecular complex was responsible for this process.^4^ However, it was not until 2002 that the term “inflammasome” was coined to describe this multi-protein complex.^3^ The first inflammasome to be identified was NACHT, LRR, and PYD domains-containing protein 1 (NLRP1) in 2002, and NLRP3 quickly followed this in 2004.^3,5^ Henceforward, many inflammasomes have been identified, each with unique immune functions and roles.

**Fig. 2:**
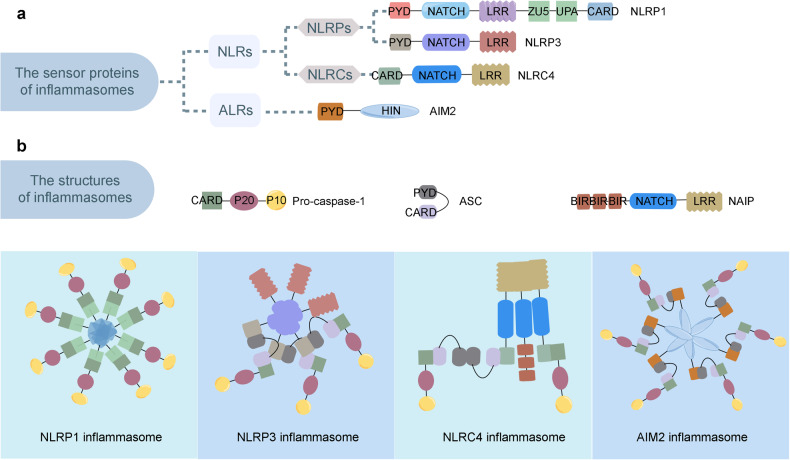
Representative structures of the inflammasome sensor proteins and the inflammasomes. **a** Representative structure of the inflammasome sensor proteins, including NLRPs (NLRP1 and NLRP3), NLRCs (NLRC4), and ALRs (AIM2). **b** Representative structure of the inflammasomes, including the NLRP1 inflammasome, the NLRP3 inflammasome, the NLRC4 inflammasome, and the AIM2 inflammasome. NLRs, nucleotide-binding domain, leucine-rich repeat-containing proteins; NLRPs, NLRs containing an N-terminal pyrin domain (PYD); NLRCs, NLRs containing a caspase activation and recruitment domain (CARD); ALRs, absent in melanoma 2 (AIM2)-like receptor; NAIP, NLR apoptosis inhibitory protein. The figure was created by FIGDRAW

Over the past few decades, there has been a growing number of different types of inflammasomes. What has ultimately allowed for distinct inflammasomes to be characterized is that each type contains unique scaffolding proteins. Most of the scaffolding proteins belong to the nucleotide-binding domain, leucine-rich repeat-containing proteins (NLRs) family, or the absent in melanoma 2-like receptors (ALRs), also known as PYRIN-HIN-200 (PYHIN) proteins family (Fig. 2a).^6–10^ NLRs play an essential role in inflammation and belong to the pattern recognition receptors (PRRs) family that sense stress signals to generate immune responses to prevent further damage.^11^ Alternatively, the HIN-200 family’s function in the mammalian innate immune system is to detect cytoplasmic stimuli in order to regulate the immune response.^10^

NLRs consist of three main components: an N-terminal effector domain, a central nucleotide-binding (NACHT) domain, and a C-terminal leucine-rich repeat (LRR) domain.^12^ Differences in the N-terminal effector domain further divide them into two subgroups: NLRs containing a pyrin domain (PYD) are members of the NLRP subgroup, and NLRs with a caspase activation and recruitment domain (CARD) are members of the NLRC subgroup. Currently, known members of the NLR family that mediate the assembly of inflammasomes include NLRP-1,3,6,7,9 and NLRC4.^3,13–17^ Upon activation, NLRs typically form an inflammasome complex with the adaptor protein ASC (apoptosis-associated speck-like protein containing CARD) connected to a downstream effector or signaling protein, such as caspase-1 or 5 (Fig. 2b).^18^

NLRs act as the sensor components of inflammasomes that recognize foreign pathogen-associated molecular patterns (PAMPs) or endogenous damage-associated molecular patterns (DAMPs). Once activated, NLRs homo-oligomerize via NACHT domains, enabling them to bind to the ASC adapter protein.^19^ The ASC adaptor protein consists of two protein-protein interaction domains: an N-terminal PYD and a C-terminal CARD.^20^ Upon recruitment to the oligomerized NLRs, ASC releases its CARD domain from the auto-inhibited conformation. The assembled ASC subsequently recruits pro-caspase via CARD-CARD interactions, thereby inducing heterodimerization, auto-cleavage, and caspase-1 activation.^21^ Active caspase-1 cleaves the intracellular pro-inflammatory cytokines, such as IL-1β and IL-18, resulting in their maturation and activation. Once activated, IL-1β and IL-18 are then secreted out of the cell where they stimulate inflammation in other cells nearby.^22^ Additionally, active caspase-1 also cleaves gasdermin D (GSDMD), releasing the N-terminal fragment of GSDMD, which induces pyroptosis and promotes further IL-1β secretion.^23^ Notably, PYHIN proteins contain a DNA-binding HIN200 domain and one or several PYD domains. This conformation allows for the formation of macromolecular complexes with other PYD-containing proteins that ultimately play a vital role in recognizing the cytosolic DNA.^9,24^ Among the PYHIN proteins, absent in melanoma 2 (AIM2) and IFI16 are the members known to be capable of caspase-1 activation.^25,26^ The C-terminal HIN200 domain of AIM2, also known as the oligonucleotide/oligosaccharide-binding domain of AIM2, acts as the sensor that recognizes DNA. Alternatively, the PYD domain of AIM2 can interact with the adapter protein ASC to induce both NF-κB and caspase-1 activation.^9^

Since their initial discovery, a growing body of research has implicated that aberrant inflammasome activity contributes to the development of multiple disorders, including metabolic, neurodegenerative, and inflammatory conditions. In recent years, there has been significant progress in identifying the mechanisms that activate inflammasomes and their role in different diseases. These discoveries have led to an increasing interest in developing new inflammasome-targeting therapies, which are presently under evaluation in numerous clinical trials. Herein, we introduce the structural basis of different inflammasomes and the mechanisms that drive inflammasome activation. We also summarize our current understanding of the various roles each inflammasome plays in the development of different diseases.

## Structure of inflammasome sensors

In the following section, we will present an overview of the well-described inflammasome structures, including the NLRP1, NLRP3, NLRC4, and AIM2 molecules.^3,9,14,27^ We will also report on the structure of molecules known to form inflammasome complexes under specific conditions, such as IFI16 (interferon-inducible protein 16), NLRC5, NLRP6, NLRP7, and NLRP9.^16,28–31^ Collectively, these studies provide insights into the molecular mechanisms of inflammasome formation and offer a basis on which to better understand the pathological consequences of various diseases on inflammasome function.

### Structure of NLRP1 inflammasomes

Multiple alternatively spliced transcript variants encoding up to 5 distinct isoforms have been found for the *Human NLRP1* gene, with the longest isoform (isoform 1) encoded by *NLRP1* transcript variant 1 (Gene ID: 22861). Isoform 1 contains several conserved domains, including the PYD (Pyrin death domain), NACHT domain, NOD2_WH (NOD2 winged helix) domain, NLRC4_HD2 (NLRC4 helical domain), LRR_RI (LRRs, ribonuclease inhibitor RI-like subfamily) domain, LRR_AMN1 (LRR [structural motif]) domain, FIIND (function to find) domain, and CARD. Compared to isoform 1, the encoded isoform 2 lacks an internal segment in the FIIND domain, isoform 3 lacks an internal segment in the LRR_RI domain, isoform 4 lacks two internal segments in the FIIND and LRR_RI domain, and isoform 5 has a shorter and distinct C-terminus. However, the functional variance of distinct isoforms has yet to be determined.

The PYD and CARD domains belong to the death domain (DD) superfamily. NMR spectroscopy analysis has shown that the structure of NLRP1-PYD differs from the DD superfamily because a flexibly disordered loop replaces its third α-helix, and this difference may influence how NLRP1 functions in protein-protein interactions.^32^ Furthermore, NLRP1 activity depends on ASC, which interacts with its C-terminal CARD domain, and on autolytic cleavage at Ser^1213^ within the FIIND, both of which are essential for NLRP1 inflammasome activity.^33^ NLRP1-CARD contains prominently charged surface patches, which can interact with the procaspase-1-CARD via a complementary charge surface.^18,34^ NLRP1-CARD forms central helical filaments, which are sufficient to induce ASC speck formation.^35,36^ Structural analysis shows that NLRP1-CARD interacts with ASC-CARD to form the filament complex via an interaction between a conserved set of interaction surfaces (Type I, II, and III).^37^ Moreover, NLRP1-FIIND is a type of ZU5-UPA domain. It contains a conserved SF/S motif and conserved glutamic acid (Glu) and histidine (His) residues adjacent to the cleavage site, which execute post-translational autocleavage and regulate the auto-processing efficiency, respectively.^38^ NLRP1-FIIND functionally reduces the NLRP1-CARD oligomerization and filament formation threshold.^36^ Alternatively, NLRP1-ZU5 inhibits NLRP1 activation by downregulating NLRP1-UPA-CARD filament formation.^38^ Additionally, mutation of His residues caused the loss of NLRP1 autocleavage.^38,39^ Isoform 2 of NLRP1 lacks exon 14 and insights from research on a disease-associated single nucleotide polymorphism (SNP) near the highly conserved distal residue His^1186^ suggest this region is important for autolytic cleavage and NLRP1 activation.^33,38^ The SNP rs11651270 (M1184V) is a common NLRP1 variant, significantly associated with asthma, that can keep NLRP1-FIIND monomeric and subsequently promote the full-length NLRP1 assembly, but is independent of autoproteolysis.^40,41^

### Structure of NLRP3 inflammasomes

*Human NLRP3* has several alternatively spliced transcript variants (Gene ID: 114548). The most commonly referred to variant for full-length NLRP3 is isoform e, which is encoded by *NLRP3* transcript variant 3 or 6. Isoform e has several conserved domains, including the PYD, FISNA (Fish-specific NATCH associated domain), NATCH domain, NOD2_WH domain, and LRR_RI domains. Compared to isoform e, isoform a contains a less conserved translational start codon and possesses two more amino acids at N-terminus; isoform b is encoded by *NLRP3* transcript variant 2, isoform c is encoded by *NLRP3* transcript variant 4, and isoform d is encoded by *NLRP3* transcript variant 5. These isoforms have shorter but different internal segments in the LRR_RI domain than isoform e, as variant 2 lacks two in-frame exons and variant 4/5 lacks one in-frame exon.

NLRP3 integrates different inflammatory stimuli and relies on distinct structural features within the N-terminus, NATCH, and LRR domains.^42^ ATP shows high binding affinity with the NLRP3-NATCH domain that mediates NLRP3 self-oligomerization, and the Walker A, B, and extended Walker B motifs are the proposed key ATP binding regions in NACHT.^43,44^ NLRP3 mutations are predicted to disrupt the structure around these ATP binding regions, changing the dynamics of the hydrogen-bond and charge interactions and enhancing their ATP binding affinity.^45^ Notably, NLRP3 assembles via PYD-PYD interactions between NLRP3 and ASC. Structural and sequence analyses have indicated that NLRP3-PYD interacts with ASC-PYD using equivalent binding interfaces composed of hydrophobic residues and charged conserved surface residues.^46–48^ NLRP3-PYD, ASC- PYD, and ASC-CARD interactions form filaments, activate NLRP3 nucleate ASC-PYD filaments, and subsequently cluster the ASC-CARD, which in turn nucleates caspase-1-CARD filaments leading to NLRP3 inflammasome activation.^21^ Moreover, there is a disulfide bond between conserved Cys^8^ and Cys^108^ that appears to be important for NLRP3 activation by sterile insults (i.e., ischemia), but not infections.^42,46^

In a resting state, an NLRP3 PYD-PYD interaction exists that forms cylindrical filaments composed of 3 major asymmetric interfaces. The most dominant interface consists of highly polar residues that mediate homomeric interactions.^49,50^ The PYD-PYD oligomerization is facilitated by the flexible linker sequence and the NLRP3-FISNA domain, and the NLRP3 conformational change is activated by K^+^ efflux.^51^ Additionally, NLRP3 forms a decamer (or dodecamer) ring cage that is held together by LRR homomeric interactions. Inside the cage, PYD forms a dimer with the NACHT domain located at the top of the ring. The acidic loop, which extends from a transition segment of LRR, mediates molecular interactions between opposing concave sites of LRRs.^50,52,53^ The ring cage structure is an inactive form of NLRP3 which localizes to membranes and is essential for NLRP3 activation and inhibition. The NLRP3 isoform lacking in-frame exons in the LRR domain cannot be activated under certain conditions.^54^ One possibility is that the alternative splicing of the LRR domain could regulate the stochastic activity of NLRs. Nevertheless, variants that arise in *NLRP3* have been implicated in several diseases.^55–59^ Therefore, further studies focused on the structure of NLRP3 are warranted to help guide further efforts in disease diagnosis and treatment.

### Structure of NLRC4 inflammasomes

The human *NLRC4* gene has 2 isoforms: a and b (Gene ID: 58484). *NLRC4* transcript variants 1, 2, and 3 encode the same protein, with the longest transcript being isoform a. Isoform a contains conserved domains, including CARD, NATCH domain, LRR_AMN1 domain, and LRR_RI domains. Compared to isoform a, isoform b is encoded by NLRC4 transcript variant 4 and it lacks the NATCH domain and an AMN1 motif in the LRR domain; thus, isoform b has a shorter LRR domain than isoform a.

The NATCH domain of NLRC4 contains a central nucleotide-binding domain (NBD) and a winged helix domain (WHD). The ADP-mediated interaction between the NBD and WHD stabilizes NLRC4’s closed conformation and the NLRC4 helical domain inhibits conserved and functional α-helixes of the NBD. The NLRC4 protein is kept in a monomeric state due to the C-terminal LRR domain blocking the NBD domain.^60^ ICE-protease Activating Factor (IPAF), also called the NLRC4, was discovered along with the finding that caspase-1 cannot be activated by full-length NLRC4, but instead by the truncated protein lacking the C-terminal LRRs.^61^ Bacterial ligands recognized by the NLR apoptosis inhibitory proteins (NAIPs) are essential for NAIP-NLRC4 inflammasome formation. Evidence suggests that NAIPs are the upstream receptors that recognize bacterial ligands, while NLRC4 functions as the downstream adaptor that congregates NAIPs for inflammasome formation.^62–64^ The NBD-associated α-helical domains of NAIP, but not the LRR domain, are believed to mediate this ligand specificity.^65^ Evidence also shows that the BIR1, pre-BIR, and HD1 domains in NAIP2 and NAIP5 are implicated as enabling the specific recognition of their respective ligands.^62,63^ Moreover, the bacterial protein PrgJ directly binds to NAIP2, forming a single ligand-bound NAIP2 molecule and sequentially triggering the formation of the NAIP2-NLRC4 inflammasome complex.^66–68^ Specifically, in response to stimuli or pathogens, NLRC4 undergoes structural remodeling and forms a wheel-like structure with a single catalytic surface. Once active, NLRC4 uses this surface to catalyze NAIP2-NLRC4 inflammasome activation via a self-propagating mechanism. This self-activation happens because the NAIP2 proteins contain a catalytic surface that matches the complementary NLRC4 oligomerization surface (the receptor surface), and together, these surfaces form the wheel-like double-ring structure of the PrgJ-NAIP2-NLRC4 complex.^68^

NAIP5-NLRC4 complexes are also large constructions containing 11 or 12 subunits that have a crucial function in the immune response to the bacterial protein flagellin. The assembly of NAIP5-NLRC4 complexes occurs in response to flagellin binding to NAIP5, which induces the recruitment of NLRC4 and subsequent formation of a disk-shaped hetero-oligomeric complex.^69^ Unliganded mouse NAIP5 recruits inactive NLRC4 via a fully exposed nucleating surface.^64^ Upon flagellin binding, the WHD of NAIP5 undergoes a steric rotation that activates NLRC4, consequently enabling NAIP5 to integrate with the NLRC4 protein, and stabilizing the NAIP5-NLRC4 complex.^64^ Flagellin-induced NAIP5-NLRC4 multimers subsequently form left- and right-handed helixes with a pitch of ∼6.5 nm and a diameter of ∼28 nm.^70^ Furthermore, NLRC4-CARD can nucleate caspase-1 assembly and activate caspase-1.^71^ The NLRC4-CARD filament is a left-handed helix consisting of 3.6 subunits per helical turn, similar to the ASC-CARD and CASP1-CARD filament.^71,72^ The upstream NLRC4-CARD and downstream CASP1-CARD interact based on the consistent helical assemblies.^73^ Mutations that have been reported influencing the NATCH or LRR domains of NLRC4 reinforce the likely pathogenicity in autoinflammatory disorders.^74–80^ Mechanistically, the p.W655C NLRC4 mutation activates the NLRC4 inflammasome via engaging 2 LRR interfaces.^78^

### Structure of AIM2 inflammasomes

The human *AIM2* gene is expressed as two isoforms (Gene ID: 9447). The longer isoform 1 is encoded by the *AIM2* variant 1, which contains two conserved domains, including the PYD and DNA-binding HIN (HIN-200/IF120x domain) domain. Alternatively, isoform 2 lacks a conserved PYD domain.

AIM2 is a member of the PYHIN family, which is characterized by an N-terminal pyrin domain that allows for the formation of multimolecular complexes via PYD-PYD interactions with other pyrin-containing proteins. Researchers found that the AIM2 PYD self-oligomerizes, and mutations on these residues could disrupt AIM2 PYD self-association (e.g., the F27G mutation).^81^ Structural analysis reveals that the AIM2 PYD domain is similar to a B-DNA cylinder, which could bind to the AIM2 HIN domain at the concave basic face, forming an autoinhibited protein complex.^82^ AIM2-PYD has a death domain fold with a distinct charge distribution and hydrophobic patches; its α2 helix contains a highly conserved lysine residue that stabilizes the short α3 helix, and the AIM2 PYD can bind the AIM2 HIN domain or the ASC PYD through the overlapping surface near its α2 helix.^34,81,83^ Moreover, different AIM2 PYD domains yield distinct conformations around the α3 helix region, as the region is highly flexible and different environments make the pre-existing conformational substrates vary.^84^ Notably, this conformational switching is believed to be important for the autoinhibition of AIM2. Researchers found that the AIM2 HIN domain could recognize double-stranded DNA (dsDNA), such as bacteria and viruses.^82^ When DNA binds, the AIM2 PYD domain separates from the HIN domain, initiating downstream signaling.^34,82^ Additional evidence demonstrated that AIM2- and ASC-PYD filaments assemble bidirectionally, whereas recognition between AIM2 and the ASC protein occurs in a head-to-tail manner and requires at least one to be oligomeric.^83^ These works indicate that the interactions between PYD-HIN and PYD-PYD are essential for AIM2’s autoinhibition and inflammasome formation. Similar to NLRP3, the helical symmetry of the AIM2-PYD filament occurs via the filaments assembled between AIM2-PYD and downstream ASC-PYD, and activated AIM2 could also nucleate the PYD filaments of ASC and induce subsequent signaling cascades.^21,85^ It should be mentioned that some research suggests that AIM2-PYD does not act as an autoinhibitory factor; instead, it couples ligand binding with oligomerization to create a structural template.^85^ AIM2-PYD drives both dsDNA binding and filament formation, and the dsDNA-binding domain of AIM2 can both oligomerize and assist filament formation forming AIM2/DNA filaments.^86^ Additionally, the results of some cryo-EM structural analyses suggest the AIM2-PYD and ASC-PYD filaments may be distinct from one another.^86^ However, research on the structure of AIM2-PYD in an inactive state has been considered controversial and requires further clarification.

### Structure of other inflammasomes

Human *IFI16* has 4 isoforms, with isoform 1 being encoded by the IFI16 variant 1 (Gene ID: 3428). Isoform 1 contains three conserved domains, including a Pyrin domain, a HIN domain (HIN-200/IF120x domain), and a Neogenin C-terminus. Compared to the IFI16 isoform 1, isoform 2, isoform 3, and isoform 4 lack the Neogenin C-terminus, but have a provisional PTZ00449 domain. IFI16 also has two HIN domains (HINa and HINb) that are comprised of a few tightly packed oligosaccharide/nucleotide-binding (OB) fold subdomains. HINa binds to the DNA backbone via loop L45 of the OB2 fold, and HINb both induces interferon (IFN)-β and binds DNA.^87–92^ IFI16 recognizes DNA non-specifically through an electrostatic attraction between the sugar-phosphate backbone of dsDNA and the positively charged residues of its HIN domain.^82^ The isolated IFI16-HIN200 domains do not oligomerize, and the non-DNA-binding PYD drives filament assembly.^85^ Moreover, IFI16 contains a highly conserved multipartite nuclear localization signal (NLS). IFI16 has been shown to detect pathogenic nuclear DNA primarily inside the nucleus, supporting the need for a functional NLS.^93^

Human *NLRC5* encodes several different isoforms (Gene ID: 84166). NLRC5 isoform 1 has the following conserved domains, including the atypical caspase recruitment domain, the NACHT domain, NLRC4_HD2, and the longest LRR domains, which include the LRR_RI and the two structural motifs LRR_RI and LRR_AMN.^94^ Compared to isoform 1, isoforms 2 to 7 and 10 have six conserved domains consistent with isoform 1, isoforms 8 and 9 have another protein phosphatase 1 regulatory subunit 42 (PPP1R42) domain, isoforms 11 to 20 lack an LRR_AMN1 domain, and isoform 21 lacks an LRR_AMN1 domain but contains a PP1R42 domain. NLRC5 is unique because it poses an unusually high number of LRRs and an atypical CARD domain. Homology modeling suggests that NLRC5 could form a homo-heptamer upon activation.^94^ Interestingly, human NLRC5 has intrinsic transcriptional activity within its N-terminal effector domain.^95^ Additionally, the NLRC5 N-terminal effector domain can interact with the downstream tandem CARD of the protein retinoic acid-inducible gene I (RIG-I).^96^ Structural analysis has also shown that NLRC5 belongs to the CARD subfamily and can be classified as an atypical CARD.^97^

Human *NLRP6* is expressed as two isoforms, with isoform 1 being longer and containing four conserved domains, including a Pyrin DD found in ASC, a NATCH domain, and two LRR_RI domains (Gene ID: 171389). Alternatively, isoform 2 lacks an LRR_RI domain but has a NOD2_WH, an NLRC4_HD2, and a PPP1R42 domain. Upon stimulation by LPS, NLRP6 binds LPS directly, subsequently dimerizes and causes global conformational changes. Following a secondary stimulation by ATP, the NLRP6 homodimer forms a linear molecular platform that recruits ASC to create a higher-level molecular structure.^98^ The PYD of NLRP6 alone is capable of self-assembling into filamentous structures that can recruit the ASC adaptor via PYD-PYD interactions.^99^ With concentration-dependent assembly, full-length NLRP6 forms filaments containing the NBD and LRR domains that surround a PYD core.^99^

The *NLRP7* gene is expressed as three isoforms, with isoform 3 being the longest and consisting of an N-terminal PYD, followed by a central NACHT domain and a C-terminal LRR domain as with all the NLRs (Gene ID: 199713). Additionally, there is a GTPase SAR1 family (Gem1) subdomain in the NACHT domain of isoform 3. NLRP7 isoforms 1 and 2 are shorter than isoform 3. Interestingly, the PYD domain of NLRP7 shows positive deviation from random coil chemical shift values, which indicates a highly α-helical structure.^100^ The NMR spectroscopic analysis demonstrates that NLRP7-PYD exhibits a six-α-helix bundle DD fold, which is different from other PYDs in that a hydrophobic cluster stabilizes helix α3 and loop α2-α3 in the NLRP7-PYD. Moreover, the electrostatic surfaces are different in NLRP7 and NLRP1 PYDs.^101^ Upon activation, the NACHT-associated domain and a small part of the LRR of one NLRP7 emerge from the protective LRR domain and interact with the formed oligomer of the NACHT domain from another NLRP7 molecule.^102^ Some missense mutations in NLRP7, such as L398R and R693W, decreased its oligomerization potential.^102^ Additionally, the NBD of NLRP7 has been shown to function as an ATP-binding domain with ATPase activity. NLRP7 inflammasome formation and activity require an intact nucleotide-binding Walker A motif in order for the NBD to effectively bind and hydrolyze nucleotides.^103^

Human *NLRP9* consists of an N-terminal PYD and a central NACHT domain, directly followed by a C-terminal LRR domain (Gene ID: 338321).^31^ Structural analysis has shown that human NLRP9-PYD has an N-terminal loop that faces toward the helical bundle’s interior, and suggests the N-terminal loop of NLRP9-PYD might regulate the inflammasome’s assembly.^104^ In contrast, another study reported that the NLRP9-PYD is monomeric and unable to nucleate ASC specks or self-polymerize, suggesting NLRP9-PYD adopts a conformation that is compatible with filament formation.^31^ These findings indicate that the formation of the NLRP9 inflammasome may differ greatly from that of other inflammasomes.

## Activation of the inflammasomes

**Fig. 3:**
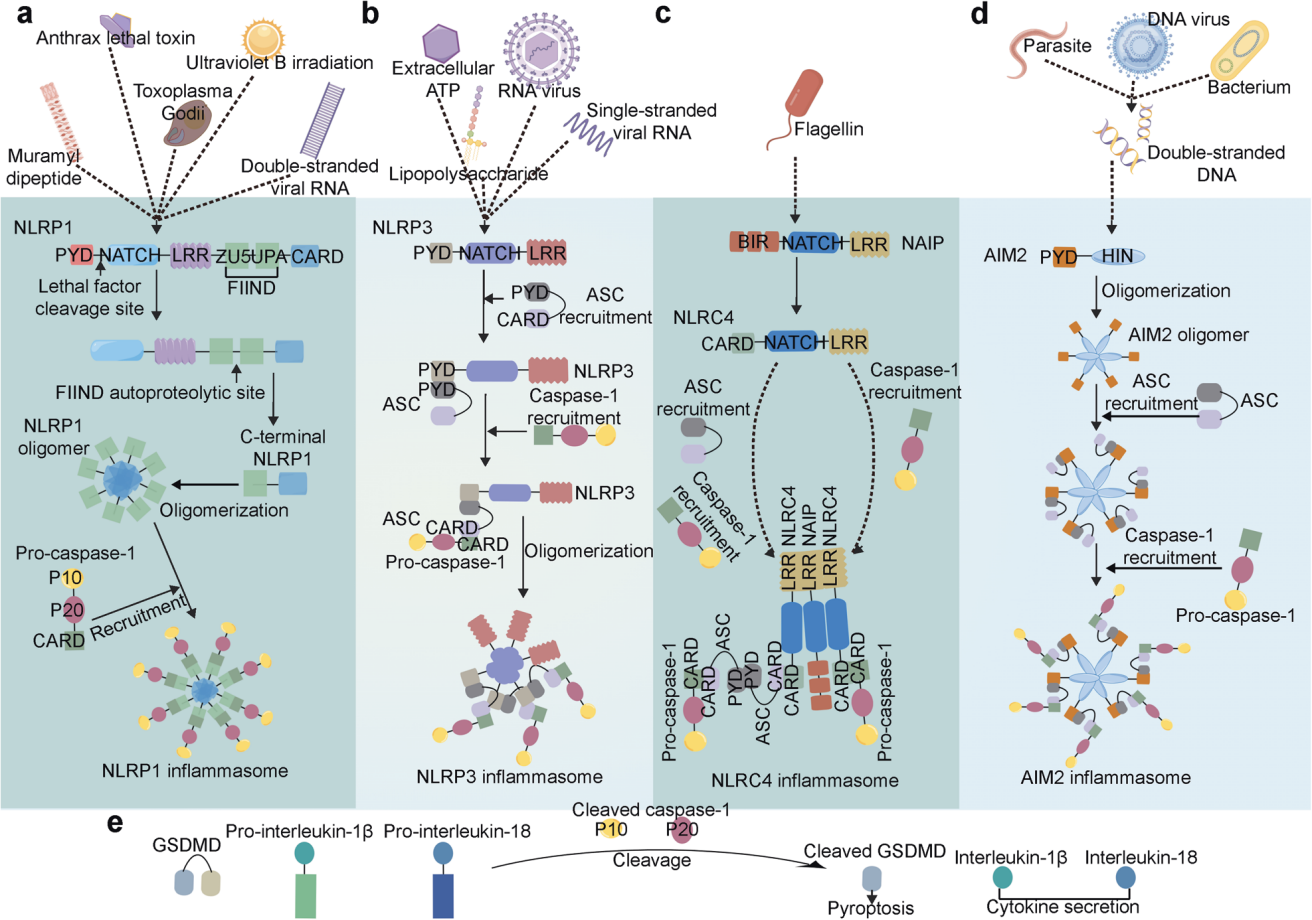
Representative pathways of the inflammasome activation. **a** Muramyl dipeptide, anthrax lethal toxin, *Toxoplasma Godii*, ultraviolet B irradiation, and double-stranded viral RNA can induce the cleavage of NLRP1, NLRP1 oligomerizes and leads to caspase-1 recruitment to form NLRP1 inflammasome. **b** Lipopolysaccharide, extracellular ATP, RNA virus, single-stranded viral RNA can activate the NLRP3, induce the ASC recruitment and following caspase-1 recruitment, and consequently form the NLRP3 inflammasome. **c** NAIP recognizes flagellin and interacts with NLRC4 to induce the ASC and caspase-1 recruitment and subsequent NLRC4 inflammasome formation. **d** Double-stranded DNA of parasite, bacterium, and DNA virus can be sensed by AIM2, then AIM2 oligomerized via its HIN domain, oligomerized AIM2 recruits ASC and caspase-1 respectively, and forms AIM2 inflammasome subsequently. **e** Inflammasomes cleave pro-caspase-1 to produce mature caspase-1 (also known as cleaved caspase-1 P10 and P20), cleaved caspase-1 cleaves GSDMD, pro-interleukin-1β, and pro-interleukin-18, cleaved GSDMD forms pyroptotic pore to execute pyroptosis, and interleukin-1β as well as interleukin-18 are released to extracellular space to regulate inflammation. The figure was created with the assistance of FIGDRAW

Upon stimulation by microbial ligands or other receptors, certain NLRs or PYHIN family members oligomerize and recruit additional components to build larger intracellular multi-protein complexes, also known as inflammasomes (Fig. 3). Although NLRP1 and NLRC4 can recruit caspase-1 directly through CARD–CARD interactions, most inflammasome sensors promote assembly by recruiting ASC via homotypic PYD‐PYD interactions (Fig. 2b).^20,21^ After recruitment, proximity-induced autoproteolytic cleavage of caspase-1 releases its catalytic subunits to form mature caspase1. Once active, caspase-1 then processes pro-IL‐1β and IL‐18 to induce the secretion of IL‐1β and IL‐18 and cleaves GSDMD to trigger pyroptosis.^22,23^

### NLRP3 inflammasome activation

To date, our most complete description of inflammasome activation is through the NLRP3 inflammasome. There are two main pathways through which NLRP3 inflammasome activation occurs, including the canonical and noncanonical pathways. Several emerging studies have focused on the molecular mechanisms that drive NLRP3 inflammasome activation and how these circumstances vary depending on the type of host cell and stimulus involved.

#### Canonical NLRP3 inflammasome activation pathway

The canonical pathway of the NLRP3 inflammasome activation begins with the induction of NLRP3, caspase-1, and pro-IL-1β expression, subsequently leading to the complex assembly comprising NLRP3, ASC, and pro-caspase-1. It includes two steps, the priming and activation steps. Stimuli, including PAMPs and DAMPs, drive both the priming and activation steps of NLRP3 inflammasome activation. Throughout this process, the inflammasome functions as a platform for attracting the pro-inflammatory cytokines, such as IL-1β and IL-18, while also facilitating their processing and maturation. Additionally, the inflammasome triggers the GSDMD cleavage, leading to the release of its N-terminal fragments and the formation of pores that facilitate pyroptosis.

During the priming phase, PAMPs and DAMPs engage with PRRs, such as NLRs and Toll-like receptors (TLRs), and this interaction facilitates the transcription and expression of NLRP3, caspase-1, and pro-IL-1β. TLRs are membrane-bound receptors that recognize PAMPs. In turn, this recognition initiates the inflammatory cascade by promoting the activation of nuclear factor-κB (NF-κB) and stimulates NLRP3 and pro-IL-1β expressions. Additionally, adequate NLRP3 levels and specific post-translational modifications are vital for NLRP3 inflammasome activation.^105^ NLRP3 is typically ubiquitinated in a resting state. The priming signals then induce NLRP3 deubiquitination, and these modifications are required for NLRP3 activation. For example, BRCC3, a deubiquitinating enzyme, has been shown to remove ubiquitin moieties from NLRP3 by directly associating with the ubiquitinated NLRP3 LRR domain in various cells, including macrophages, 293 T, and NG5 cells.^106^ Abraxas brother 1 (ABRO1) also modulates NLRP3 deubiquitination and assists BRCC3 to activate NLRP3 inflammasome.^107^ Intriguingly, phosphorylation of NLRP3 at distinct amino acid sites yields the different effects. NLRP3 phosphorylation at Ser295 or Ser5 suppresses the assembly of the NLRP3 inflammasome platform, thereby inhibiting inflammasome activation.^108^ In contrast, phosphorylation of human NLRP3 at Ser198 is essential for NLRP3 deubiquitination and the subsequent NLRP3 inflammasome activation.^109,110^ Collectively, these findings suggest the priming step in NLRP3 inflammasome activation is highly regulated by post-translational modifications of NLRP3.

The NLRP3 inflammasome activation phase is regulated by many factors that ultimately help dictate how a given immune cell responds to the pathogen. For example, activation of the ATP-gated ion channel P2X purinoceptor 7 (P2X7) leads to K^+^ efflux and Ca^2+^ influx which has been shown to help trigger NLRP3 inflammasome activation by disrupting mitochondrial ion balance and subsequent mitochondrial reactive oxygen species (mROS) generation.^111^ Cl^−^ efflux contributes to the NLRP3 inflammasome activation through a distinct different mechanism, whereby K^+^ efflux induces the oligomerization of NLRP3 while Cl^−^ efflux promotes the polymerization of ASC.^112^ These findings demonstrate that ion flux plays a crucial role in NLRP3 inflammasome activation, indicating their modulation may serve as a potential therapeutic target. Dysregulation of various organelles is also involved in NLRP3 activation, encompassing disturbances in lysosomes, mitochondria dysfunction, and disintegration of the trans-Golgi apparatus. The lysosomotropic dipeptide Leu-Leu-Ome promotes lysosomal rupture and induces an ion exchange (K^+^ efflux and Ca^2+^ influx) to allow the activation of the NLRP3 inflammasome.^113^ Mitophagy clears the impaired mitochondria and suppresses the mROS release, which serves as the activator of NLRP3. In addition, NLRP3 activation facilitates the disassembly of the trans-Golgi network into dispersed vesicles, which recruit NLRP3, followed by the promotion of ASC polymerization and the subsequent downstream signaling that leads to NLRP3 activation.^114^ Cannabinoid receptor 1 (CB1R) has also been shown to help regulate activation of the NLRP3 inflammasome. After internalization, CB1R interacts with NLRP3, caspase 1, and GSDMD proteins to inhibit the degradation of NLRP3 inflammasome.^115^ However, whether CB1R is involved in the lysosome disruption is still unclear.

#### Non-canonical NLRP3 inflammasome activation pathway

The non-canonical NLRP3 inflammasome pathway involves the activation of “non-canonical” caspases, including caspase-4, caspase-5, and caspase-11. The non-canonical pathway is also activated by direct cytosolic stimuli and is most closely associated with its significance in inflammatory disorders. Unlike the canonical pathway, caspase-4 has been shown to act as the major caspase involved in non-canonical NLRP3 inflammasome activation and it has been demonstrated that Gram-negative bacterial infections induce the non-canonical pathway, which results in cellular damage and death via pyroptosis.^116,117^

Non-canonical activation of the NLRP3 inflammasome is induced by Gram-negative bacterial infections.^116^ PAMPs and DAMPs interact with TLRs to activate NF-κB and promote NLRP3 transcription. Recent advances have discovered that in mice caspase-11 (analogous to caspase-4 in humans) was activated in the LPS signaling pathway rather than caspase-1.^118^ Caspase-11 deficiency mice exhibited attenuated IL-1β production; however, caspase-11-/- macrophages exhibited normal IL-1β production in response to stimuli, suggesting that although LPS is involved in the canonical activation of the NLRP3 inflammasome,^119^ non-canonical inflammasome activation is dependent on caspase-11.^116^ LPS stimulates TLR4, and TLR4 signaling induces activation of mitogen-activated protein kinases (MAPKs), NF-κB, and interferon regulatory factors (IRFs).^120^ Subsequently, these events promote the transcription of IL-1β, IL-18, and NLRP3. Elevated IRF-3 and IRF-7 then form a complex which induces the expression of IFN-α/β.^121^ The binding of IFN-α/β to the IFN-α/β receptor results in activation of the JAK/STAT pathway and, consequently, upregulating the transcription of caspase-11.^122,123^ Active caspase-11 triggers pyroptosis by cleaving GSDMD, resulting in the release of HMGB1 and IL-1α.^124^ Additionally, researchers have identified a hypotonicity-induced NLRP3 activation mechanism, which demonstrated that low osmolarities can trigger a downregulation in intracellular Cl^−^ concentrations that are sufficient to activate the NLRP3 inflammasome.^125^

However, it should be mentioned that certain details of the non-canonical pathway remain controversial. For instance, it was demonstrated that NLRP3 inflammasome activation can occur without the priming signal in human monocytes,^126^ and there is also evidence that a single stimulus can provide both the priming and activating signals.^127^ Advanced studies are needed to clarify this issue further. Ultimately, both the canonical and non-canonical pathways of NLRP3 inflammasome activation lead to pyroptosis through a mechanism that was triggered by GSDMD cleavage. Gasdermin E (GSDME) has also been identified as a downstream molecular of NLRP3-induced pyroptotic pathway.^128^ However, the levels of endogenous GSDME are relatively low and its function is still not fully understood. Thus, GSDMD has been recognized as a major executor that mediates pyroptotic cell death upon NLRP3 inflammasome activation.

### NLRP1 inflammasome activation

Tschopp et al. discovered and described the first inflammasome-forming sensor, human NLRP1 in their landmark 2002 paper.^3^ Although it was the first to be discovered, the activation of NLRP1 has remained unclear despite the continued and ongoing research that has been conducted to elucidate its underlying mechanism. As the domain structure differs between NLRP1 and NLRP3, there are certain key differences between the components of NLRP3 and NLRP1 inflammasomes that we will discuss below.

Diverse bacterial and protozoan toxins can activate the NLRP1 inflammasome, including ultraviolet B irradiation, double-stranded viral RNA, viral proteases, the bacterial cell wall component muramyl dipeptide, and LeTx exposure.^129,130^ There are also several ways in which pathogens can activate the NLRP1 inflammasome. First of all, in response to enterovirus 3 C cysteine proteases or the anthrax lethal factor protease, cleavage of NLRP1 near its N-terminal PYRIN domain occurs, allowing the N-terminal NLRP1 to be sent to the proteasome for degradation^131^ and consequently inducing C-terminal CARD domain oligomerization and the activation of caspase-1.^35^ Additionally, long double-stranded RNA (dsRNA) or RNA-positive (+RNA) strands could bind to the NACHT-LRR domain of NLRP1 to activate NLRP1.^130^ Upon activation, NLRP1 oligomerizes and leads to caspase-1 activation and IL-1β secretion.^132^ Additionally, NLRP1 also cleaves pro-caspase-5, which could promote IL-1β in human keratinocytes.^133^ K^+^ efflux has also been implicated in NLRP1 activation.^134^ Furthermore, studies have also shown that 3C-like protease can inactivate the GSDMD, enabling NLRP1-induced caspase-3 activation to drive Gasdermin E-dependent pyroptosis.^135^

### NLRC4 inflammasome activation

NLRC4 belongs to the NLRC family, which plays a vital role in the immune response to bacterial pathogens. Similar to other inflammasomes, transcriptional and post-transcriptional mechanisms tightly regulate NLRC4 activation.^61^ However, ligand binding and phosphorylation are the most well-described regulatory mechanisms of NLRC4 inflammasome activation. Regardless of the modifications involved, activating P53 through genotoxic stress or pro-inflammatory stimuli leads to the upregulation of NLRC4 expression.

Gram-negative bacteria with type III or IV secretion systems activate the NLRC4 inflammasome.^136–138^ Cytoplasmic injection of bacterial components from these types of bacteria may be able to trigger NLRC4 activation directly, and flagellin localization in the cytosol is sufficient to activate caspase-1 in an NLRC4-dependent manner.^139,140^ Other PAMPs may modulate NLRC4, as both flagellin-dependent and flagellin-independent mechanisms are involved in the activation of NLRC4 by *P. aeruginosa*.^141,142^ Since NLRC4 does not directly interact with an activating ligand, NLRC4 may sense cytosolic PAMPs through a common pathway, similar to the pathways proposed in NLRP3 activation. NLRC4 phosphorylation by PKCδ is essential for the NLRC4 inflammasome activation.^143^ Unlike NLRP3, high extracellular K^+^ does not inhibit NLRC4 activity, indicating NLRC4 is not an ionic flux sensor.^144^ Additionally, NLRC4 must collaborate with another NLR, NAIP, to protect against this pathogen.^63,145^ NAIPs are the upstream receptors that recognize bacterial ligands which then mediate NLRC4 inflammasome formation. NAIPs can interact with NLRC4 and induce NLRC4 oligomerization upon bacterial ligand binding. As NLRC4 lacks the PYD domain, NLRC4 may recruit a PYD-containing protein (such as an NLRP3) to react to bacterial infections.^146^ Moreover, NLRC4 contains a CARD domain, which suggests direct interactions with procaspase-1.^34^ ASC is not necessary for NLRC4-dependent caspase-1 activation in response to *L. pneumophila*. At the same time, ASC is required for the maximal response in bacterial-induced caspase-1 activation.^147,148^ These findings demonstrate that ASC and NAIP are crucial for NLRC4 inflammasome activation, although the exact mechanisms remain unclear and need further investigation.

### AIM2 inflammasome activation

AIM2 is a PYHIN family member that plays a vital role in recognizing cytosolic DNA. As the first non-NLR family member to be identified as forming an inflammasome scaffold, AIM2 can recruit ASC and activate caspase-1-dependent IL-1β maturation. The AIM2 inflammasome protects against pathogens, like *Francisella tularensis* and *Listeria monocytogene*, by sensing cytosolic dsDNA.^149,150^ As mentioned above, oligomerization of the AIM2 inflammasome is mediated by binding between sites clustered on ligands and the C-terminal HIN domain of AIM2, but not by the central oligomerization domain (as was the case for the NACHT domain in NLRs). AIM2, ASC, and caspase-1 form the AIM2 inflammasome. As for NLRP3, AIM2 has a PYD domain that interacts with ASC through homotypic PYD-PYD interactions., which enables pro-caspase-1 recruitment via the ASC CARD domain. Subsequently, activation of caspase-1 promotes the maturation and secretion of pro-inflammatory cytokines (such as IL-1β and IL-18). Additionally, AIM2 drives a form of inflammatory signaling and cell death, known as PANoptosis, by regulating the innate immune sensors ZBP1 and pyrin.^151^ AIM2 has also been shown to have permissive ligand requirements, as bacteria and cytosolic dsDNA from viruses or the host can activate AIM2.^152^ As a result, it has been suggested that AIM2 is involved in self-DNA-induced autoimmune responses in systemic lupus erythematosus. Additional studies to further disambiguate the viral dsDNA and self-DNA pathways are needed.

### IFI16, NLRC5, NLRP6, NLRP7, and NLRP9 inflammasome activation

IFI16 is another member of the PYHIN family that can form an atypical inflammasome. IFI16 expression has been found in myeloid precursor cells, mature lymphocytes, peripheral blood monocytes, T cells, and epithelial cells.^153–155^ Unlike AIM2, IFI16 is primarily found in the nuclei of resting cells and serves to recognize viruses that enter the nuclei. Several pathogens are known to be recognized by IFI16, including the Kaposi sarcoma-associated herpesvirus (KSHV) and the influenza A virus (IAV).^156–158^ IFI16 migrates to the cytoplasm from the nucleus upon activation, forming nuclear and cytosolic inflammasomes containing IFI16, ASC, and caspase-1.^156^ Subsequently, the IFI16 inflammasome induces caspase-1 activation and IL-1β cleavage. KSHV-induced IL-1β and IL-6 expressions are dependent on IFI16 and ASC expression,^156^ suggesting that the IFI16 inflammasome, but not other inflammasomes, initiates the responses to KSHV infection. Additionally, IFI16 binds to viral DNA and subsequently facilitates IFN-β production via a direct interaction between IFI16 and STING.^159^ It is evidenced that IFI16 is a unique PRR that plays dual roles in the cytoplasm and nucleus. Moreover, the existence of both nuclear and cytosolic inflammasomes indicates that the innate immune system applies a multifaceted approach to detect intracellular pathogens.

NLRC5, like NLRC4, is also vital in antibacterial defenses. NLRC5 is expressed both in the cytoplasm and nucleus of cells, and NLRC5 can regulate MHC class I gene expression. It is widely accepted that NLRC5 regulates gene expression within the nucleus, but its function within the cytoplasm is less clear and needs to be understood. In monocytes, NLRC5 plays a vital role in mediating caspase-1 activation and IL-1β secretion in response to infections from *Escherichia coli*, *S. aureus*, and *Shigella flexneri*, or upon stimulation by TLR ligands.^29^ Intriguingly, NLRP3 agonists can trigger IL-1β secretion via NLRC5. ASC and NLRP3 were also found to interact physically with NLRC5, with intact NACHT domains being required for NLRC5 to bind to NLRP3. The co-expression of ASC, pro-caspase-1, pro-IL-1β, NLRC5, and NLRP3 in HEK293T cells causes IL-1β cleavage.^29^ Interestingly, TLR ligands and NLRP3 agonists appear to have no effect on cytokine production in the absence of NLRC5, whereas the co-expression of NLRC5, pro-caspase-1, and pro-IL-1β without NLRP3 induces the cleavage of IL-1β.^160^ These data suggested that NLRC5 appears to be able to form a functional inflammasome, although it remains to be determined if NLRC5 can form an inflammasome complex independently.

NLRP6 inflammasomes have previously been reported to regulate intestinal microbiota in mice.^15^ There is also evidence that NLRP6 can form inflammasomes in vitro. For example, in a study where NLRP6 and ASC were expressed in 293T cells, NLRP6 was recruited into speck-like structures within the ASC. Moreover, transfecting plasmids encoding NLRP6, ASC, and pro-caspase-1 into COS-7L cells induces IL-1β secretion.^161^ Further evidence of NLRP6 inflammasomes comes from studies showing that NLRP6-deficient mice developed enhanced dextran sulfate sodium (DSS)-induced colitis, like that of ASC- and IL-18-deficient mice. Intriguingly, one study reported that housing wild-type mice and NLRP6-deficient mice together enhanced disease severity in wild-type mice, which was interpreted as evidence that microbiota can act as a driving factor of enhanced colitis in an NLRP6-dependent manner. In addition, IL-18 production was crucial for maintaining intestinal homeostasis. It was also hypothesized that unknown ligands might activate the NLRP6 inflammasome, ultimately leading to the IL-18 maturation, preventing dysbiosis, and regulating microbiota through an unknown mechanism. Further insight into the function of NLRP6 inflammasome in intestinal homeostasis was gained through studies on mucus production in mice challenged with an enteric pathogen.^162^ There was a significant decrease in mucus thickness in mice lacking NLRP6, ASC, or caspase-1, which increased bacterial adherence and dissemination in *C. rodentium*-infected mice. The NLRP6 inflammasome is clearly involved in protecting the intestinal barrier and regulating dysbiosis; however, how intestinal ligands activate the NLRP6 inflammasome remains unclear.

NLRP7 inflammasome activation was reportedly identified in human macrophages upon exposure to microbial acylated lipopeptides.^16^ Various bacterial acylated lipopeptides, as well as TLR2 agonists, could activate NLRP7-inducing caspase-1-mediated IL-1β production. Additionally, the activation of TLR2 in response to acylated lipopeptides is believed to be reliant on the maturation of IL-1β through NLRP7 and required for the transcription of chemokines, pro-IL-1β, and pro-IL-18. The transcription of these signaling molecules, in turn, acts as the priming step for NLRP7 inflammasome activation. By activating NLRP7, caspase-1 becomes enzymatically active, leading to IL-1β and IL-18 maturation, which subsequently restricts intracellular bacterial replication. However, caspase-1-independent IL-6 or tumor necrosis factor-α (TNF-α) secretions are not observed following NLRP7 inflammasome activation. Additionally, some in vitro studies have found that NLRP7 is a negative regulator of inflammation. For instance, NLRP7 directly interacts with pro-caspase-1 and inhibits IL-1β maturation in transiently transfected HEK293 cells.^163^ The same situation occurred in THP-1 cells, where NLRP7 levels were increased upon LPS or IL-1β stimulation. Specifically, NLRP7-expressing THP-1 cells secreted less IL-1β than empty vector-transfected cells when treated with LPS. Consistently, patients with a hydatidiform mole during pregnancy who have NLRP7 mutations and rare variants showed low levels of IL-1β and TNF secretion in response to LPS.^164^ Mechanistically, the PYD is critical for inhibiting IL-1β processing, such that protein-truncating mutations after the PYD abolish IL-1β inhibition. NLRP7 co-localizes with Golgi and microtubule-organizing centers in peripheral blood mononuclear cells, which indicates that NLRP7 also influences IL-1β and TNF secretion via cytokine trafficking in these cells. The exact role of NLRP7 in innate immunity remains unclear. Furthermore, it is unknown whether NLRP7 inflammasomes can be formed in other cell types or if other ligands can activate NLRP7. These questions may serve as exciting areas of research in the near future.

A recent study has suggested that NLRP9 could initiate inflammasome formation upon short dsRNA stimulation. NLRP9b acts as a sensor detecting rotavirus in the intestine. Interestingly, NLRP9b did not directly bind viral RNA; instead, the RNA helicase DHX9 acted as a direct RNA-binding protein that mediates viral recognition by NLRP9.^17^ However, the mechanism by which DHX9 differentiates between the viral and host RNA is still unclear. Interestingly, NLRP9b is mainly expressed in intestinal epithelial cells but not the neighboring immune cells. NLRP9b conditional KO mice showed higher susceptibility to rotavirus infection, suggesting that NLRP9b exerts a protective function in intestinal epithelial cells. Upon rotavirus infection, NLRP9b forms inflammasomes with ASC and caspase-1 to trigger the maturation of IL-18 and GSDMD-induced pyroptosis in mice.^17^ NLRP9b-, ASC-, or caspase1- deficient mice exhibit elevated viral loads and pathological symptoms than wild-type mice, suggesting that NLRP9b could initiate inflammasome formation upon rotavirus infection. Moreover, GSDMD-deficient mice are more vulnerable to rotavirus infection, suggesting that rotavirus clearance requires GSDMD. Human NLRP9 is also capable of binding to rotavirus RNA. However, it remains unclear whether human NLRP9 performs the same functions as the murine NLRP9. Further investigations are warranted to determine the exact molecular mechanisms underlying human NLRP9 inflammasome formation.

These studies provide a better understanding of innate immunity and inflammasomes, and suggest there may be new potential therapeutic targets for diseases associated with aberrant inflammasome activity. These studies also reveal that inflammasomes are expressed in various types of cells and respond to diverse pathogens in both the cytoplasm and nucleus. Variations in the mechanisms that regulate inflammasome activation reported by these studies could be attributed to several factors, including inherent differences in cell types, the expression levels of inflammasomes, and the types of stimuli used to challenge cells. Further investigations focused on the precise role that inflammasomes play in modulating the innate immune signaling pathways should be conducted in the relevant systems.

## Roles of the inflammasomes in various diseases

**Fig. 4:**
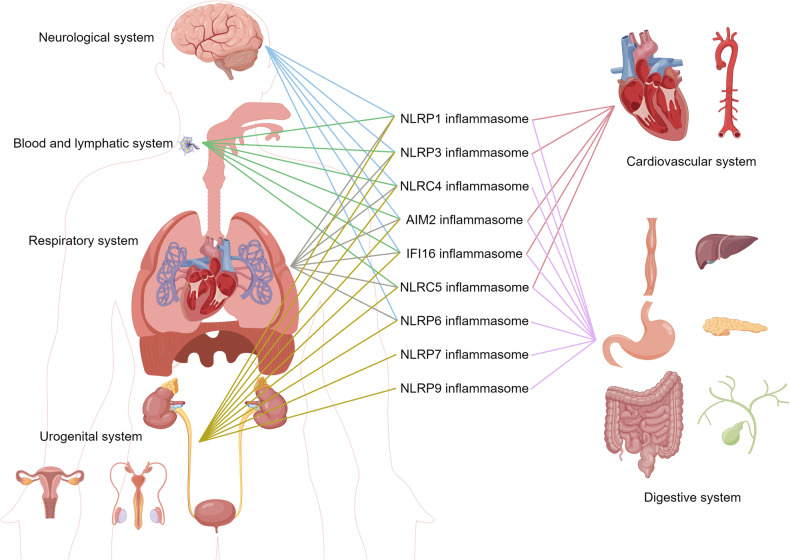
Different inflammasomes contribute to diseases of different systems in the human body. The figure was created with the assistance of FIGDRAW

There is a strong correlation between inflammasomes and various autoimmune and autoinflammatory diseases, including cardiovascular diseases, neurodegenerative diseases, and metabolic disorders (Fig. 4). Similarly, as our understanding of inflammasomes has grown over the past few decades, it has become increasingly clear that inflammasomes play either causative or contributing roles in the initiation and progression of various diseases. Here, we provide an overview of the potential roles those inflammasomes may play in different diseases.

### Cardiovascular disorders

Inflammation plays a key role in the development of cardiovascular disorders, and aberrant inflammasome activity has been implicated in several of these conditions, including atherosclerosis. Atherosclerosis is a chronic disease characterized by the progressive hardening or narrowing of arterial vessels that can lead to heart attacks and strokes.^165,166^ In atherosclerosis, high quantities of cholesterol and white blood cells clog the arterial wall, preventing oxygen-rich blood from reaching the organs.^167–169^ Compared to disease-free arterial tissues, atherosclerotic plaques contain higher levels of IL-18 and IL-18 receptors. Inflammasome activation leads to elevated IL-18 production, which may contribute to the pathology of atherosclerosis.^170^ For example, mice lacking Apolipoprotein E (ApoE) develop atherosclerosis spontaneously, and atherosclerotic plaques are more unstable when IL-18 levels are high, whereas IL-18 deficiency results in smaller lesions.^171^ Elevation of free fatty acids (FFAs) and low-density lipoprotein (LDL) in human blood can induce pro-IL-1β production via TLRs.^172^ The cell surface receptor CD36 promotes oxidized LDL (ox-LDL) internalization and cholesterol crystallization, and these cholesterol crystals activate the NLRP3 inflammasome in vitro via phagolysosomal damage.^173^ This data indicates that FFAs or LDL can provide the priming or activation signal for inflammasomes. In macrophages, cholesterol activates the NLRP3 inflammasome and mediates IL-1β release in a Cathepsin B-dependent manner.^174^ In LDL receptor knock-out mice, transplantation of bone marrow from NLRP3-/-, ASC-/-, or IL-1β-/- mice showed decreased atherosclerosis.^170^ Similarly, the size of atherosclerotic lesions in ApoE-deficient mice is significantly reduced by IL-1β inactivation.^175,176^ These findings are promising and suggest additional studies should be conducted to clarify the exact mechanism of inflammasome activation in atherosclerosis, as well as the contribution of IL-1β to atherogenesis.

Hypertension can cause myocardial hypertrophy and fibrosis, leading to the development of heart failure. Hypertension-induced cardiac or vascular upregulation of NLRP3 and IL-1β has been observed in different animal models, such as spontaneously hypertensive rats, decompensated right ventricular hypertrophy rats, and deoxycorticosterone acetate-induced hypertensive mice.^163,177,178^ Interestingly, a transverse aortic constriction (TAC) was also found to increase NLRP3 and caspase-1 activity in cardiomyocytes, but not in non-cardiomyocytes.^179^ This suggested that the original site of NLRP3 inflammasome activation may be in cardiomyocytes. However, it remains unclear how inflammasomes activate without ischemic damage or cell death. There is evidence that activation of the NLRP3 inflammasome is mediated by increased Ca2^+^/calmodulin-dependent protein kinase II δ (CaMKIIδ) activity in response to pressure overloads.^179^ Using cardiomyocyte-specific CaMKIIδ-KO (CKO) mice, researchers observed that caspase-1 activity was attenuated, and IL-1β and IL-18 levels were reduced in TAC-treated CKO mice.^179^ Additionally, diabetes mellitus may lead to diabetic cardiomyopathy, in which NLRP3 activation in a Cathepsin B-dependent manner could aggravate the condition by promoting pyroptosis.^180^ These effects may be intensified by glucose, which is a potential stimulus for NLRP3.^181^ Altogether, these findings suggest that therapies targeting the NLRP3 inflammasome may help to prevent cardiac remodeling and heart failure.

The NLRP3 inflammasome has also been implicated in the development of atrial fibrillation. In atrial cardiomyocytes from patients with atrial fibrillation, NLRP3 inflammasome activity was increased.^182^ Mice expressing constitutively active NLRP3 in their cardiomyocytes (cardiomyocyte-KI) showed spontaneous premature atrial contractions; using MCC950 to inhibit NLRP3 blunted the spontaneous premature atrial contractions.^182^ Cardiomyocyte-KI mice exhibited larger atria, electrical remodeling, and abnormal spontaneous Ca2^+^ release patterns from the sarcoplasmic reticulum, which were prevented by the knockdown of NLRP3 in cardiomyocytes. These findings suggest that targeting the NLRP3 inflammasome could be a new therapy for atrial fibrillation.

There is evidence that dilated cardiomyopathy is accompanied by an inflammatory component that plays an important role in its pathogenesis. For instance, there is a clinical correlation between circulating levels of NLRP3 inflammasome and cardiac function, as well as between the NT-pro BNP levels and the cumulative rehospitalization rate in patients with dilatated cardiomyopathy.^183,184^ NLRP3 activation also occurs in a time-dependent manner in response to ischemia.^185^ Ischemic cells release DAMPs and alarmins, which strongly stimulate the NLRP3 inflammasome. During the healing phase, ASC aggregates are most prevalent in cardiomyocytes and fibroblasts.^186,187^ Furthermore, patients with acute myocarditis have been found to have NLRP3 inflammasomes in their endomyocardium.^188^ CVB3, a common virus causing myocarditis, increases caspase-1, ASC, and IL-1β expression in infected mice by altering NLRP3 activation.^189^ Mechanistically, Cathepsin B mediates both inflammasome activation and pyroptosis in experimental CVB3-induced myocarditis.^190^

The NLRP3 inflammasome also regulates the initiation and propagation of another cardiovascular disorder: Venous thromboembolism (VTE). Elevated NRLP3 activity, indicated by high caspase-1, IL-1β, IL-6, or C-reactive protein levels, was observed in patients with VTE.^191^ Hypoxia and reduced blood flow induce NLRP3 activation and elevated levels of caspase-1 and IL-1β following experimental venous thrombosis.^192^ Experimental intervention studies have also found that genetic deletion of NLRP3, caspase-1, or GSDMD, and inhibition of caspase-1 or IL-1β has been shown to ameliorate venous thrombosis.^193^ Altogether, developing novel therapeutics against VTE may be possible by selectively targeting the NLRP3 inflammasome and maximizing the benefits of anticoagulation. In summary, there is considerable evidence that inflammasomes play either causative or contributing roles in the development of several cardiovascular diseases. This data suggests that inflammasomes, caspase-1, and IL-1β may be promising therapeutic targets for cardiovascular diseases.

Several commonly used medications like anti-tumor drugs (i.e. doxorubicin) and antipsychotic drugs are reported to have significant cardiotoxic effects.^194,195^ Moreover, a recent study found that Sirtuin 3 alleviates the doxorubicin-induced cardiotoxicity by inhibiting the activation of the NLRP3 inflammasomes.^196^ Antipsychotic cardiotoxicity was predominantly mediated by CB1R translocation-induced NLRP3 inflammasome stabilization and subsequent pyroptotic cell death.^115^ These findings indicate that inflammasome activation may act as a mediator of drug-induced toxicity. As such, targeting the inflammasome signaling pathway has the potential to relieve the cardiotoxic effects of anti-tumor or antipsychotic drugs.

### Neurological disorders

NLRP3 is the first inflammasome to have been studied in the central nervous system (CNS), and while it is predominantly located in microglia, it is also expressed in neurons, astrocytes, and oligodendrocytes.^197–200^ NLRP3 inflammasomes play a crucial role in several cerebral pathologies, including Alzheimer’s disease (AD), Parkinson’s disease (PD), amyotrophic lateral sclerosis (ALS), multiple sclerosis (MS), and CNS infections.^201^ NLRP3 has two major isoforms, including the full-length NLRP3 and the one without exon 5.^54^ The full-length NLRP3 protein functions effectively, while the NLRP3 isoform without exon 5 is a non-functional variant that cannot be activated. The functional NLRP3 proteins are involved in neuroinflammation of neurodegenerative diseases. However, the exact functions of full-length NLRP3 and the exon 5-lacking in neurodegenerative disorders are still unclear. Other inflammasomes, including NLRP1, NLRC4, and AIM2, have also been implicated in some neurological disorders.^202–204^ AIM2 has been detected in most cells of the CNS, including neurons, microglia, and brain endothelial cells. On the other hand, NLRP1 inflammasomes have been observed predominantly in neurons, whereas NLRC4 expression has been found mainly in microglia and astrocytes. Furthermore, hallmarks of neurodegenerative disease such as amyloid- β (Aβ), α- synuclein (α- Syn), and transactive response DNA-binding protein 43 (TDP43) can act as immune stimulatory molecular patterns in the CNS.

AD is the most common neurodegenerative disorder.^205^ It is characterized by the formation of neuritic plaques and neurofibrillary tangles (NFTs) in the brain. Neuritic plaques are caused by the accumulation of Aβ, whereas NFTs are a result of hyperphosphorylated tau inside neurons.^206^ Aβ is primarily produced within neurons and is then released from the brain into the CSF and blood vessels.^207^ Upon exceeding a critical threshold, Aβ forms oligomers, fibrils, and deposits in neuritic plaques, which can act as DAMPs to activate NLRP3 inflammasomes.^208,209^ Fibrillar Aβ-induced microglial IL-1β release occurs in an NLRP3- and ASC-dependent manner.^210,211^ In addition, soluble and oligomeric Aβ peptides are equally as potent in inducing CD36-mediated NLRP3 inflammasome activation and IL-1β production.^173^ NLRP3 activation is further regulated by autophagy-mediated autophagy, as deficiencies in the cellular autophagy-related protein 7 (ATG7) were found to increase caspase-1 cleavage and IL-1β release in microglia.^212^ Another study found that cell media collected from Aβ-treated microglia was neurotoxic, and this effect was more pronounced in ATG7-deficient microglia, indicating that well-controlled microglial inflammasome activation could limit neuronal destruction. Studies have also shown that NLRP3 inflammasome functions in astrocytes, and astrocytes can release IL-1β in an ASC-dependent manner upon uptake of Aβ.^213^ However, the NLRC4 inflammasome was also found in astrocytes and could promote IL-1β maturation.^214^ Whether different inflammasomes act synergistically or independently needs further investigation. In an in vivo uptake assessment, amyloid precursor protein (APP)/presenilin 1 (PS1)/NLRP3-knockout mice (mice generated from the cross of APP/PS1 mice and NLRP3-knockout mice) showed evidence of Aβ phagocytosis and an increased phagocytic clearance capacity.^215^ Insulin-degrading enzyme (IDE) expression was also increased in brain lysates obtained from APP/PS1/NLRP3-knockout mice.^215^ IDE has been shown to degrade extracellular Aβ, indicating that NLRP3 plays a role in balancing the cerebral Aβ load. Additionally, synaptic loss occurs in AD brains, and blocking NLRP3-associated signaling protects against neuronal spine loss in APP/PS1 mice. Furthermore, the ASC speck formation is another feature of inflammasome activation, and upon release, the ASC specks bind quickly to Aβ peptides.^216^ Aβ was also shown to bind with ASC in brain samples from AD and APP/PS1 mice.^216^ Moreover, ASC specks can seed Aβ deposition in APP/PS1 mice.^217^ These findings suggest that the release of ASC specks may contribute to early Aβ deposition and the pathogenesis of AD. A recent study also found that susceptibility to inflammasome activation was correlated with a higher likelihood of cognitive deficits in AD patients.^218^ Additionally, AIM2 and NLRP1 inflammasomes in particular have been implicated in AD as Alzheimer’s patients have increased NLRP1 expression in their brains. Neurons in APP/PS1 mice showed an upregulation of NLRP1 levels.^219^ Also, NLRP1 reduced the number of apoptotic neurons but had no effect on overall Aβ deposition in this model. In addition, AIM2 is believed to play a vital role in AD pathogenesis, as microglial activation was attenuated, and IL-6 and IL-18 levels were increased when AIM2 was knocked out in 5XFAD mice. However, both open-field behavior and spatial memory performance were not improved,^220^ suggesting the AIM2 inflammasome may not directly affect cognition or memory formation.

Research suggests that α-Syn could also trigger inflammasome activation In PD. α-Syn can induce NLRP3 inflammasome activation and IL-1β release in both monocytes and microglia in a cathepsin B- and caspase-1-dependent manner.^221,222^ TLR2 inhibitors can block this α-Syn induced IL-1β release in human monocytes.^223^ This suggests that TLR2 may mediate the signaling pathway by which α-Syn induces NLRP3 inflammasome activation. However, additional data is needed to clarify this mechanism and its role in PD. In MPTP-induced PD mice and human microglia, dopamine inhibits the activation of microglial NLRP3 inflammasome through signaling via DRD1 and DRD2, leading to the ubiquitination and subsequent degradation of NLRP3.^224^ Similarly, inhibiting microglial NLRP3 inflammasome activation significantly reduced dopaminergic neurodegeneration and ameliorated motor deficits in the MPTP-treated mice, although the mechanism behind this remains unknown.^225^ Neuronal NLRP3 has also been implicated in PD pathogenesis. The parkin protein encoded by *PRKN* functions as an E3 ubiquitin ligase, and PRKN mutations lead to monogenic PD.^226^ In dopaminergic neurons, reducing parkin activity induces spontaneous activation of the NLRP3 inflammasomes, and parkin inhibits this activation by ubiquitinating NLRP3.^227^ These findings suggest that both neuronal and microglial NLRP3 inflammasomes play a role in the pathogenesis of PD; whether the two work synergistically remains to be determined.

ALS is a neurodegenerative disease that gradually destroys upper and lower motor neurons, leading to the atrophy and paralysis of voluntary muscles. Superoxide dismutase (SOD1) and TDP43 are key molecules at high risk of genetic mutations that cause familial ALS.^209,228^ There has been evidence that the NLRP3 inflammasome is activated in the brains and spinal cord of sporadic ALS patients as well as in ALS mouse models. Bioptic samples taken from the spinal cord of ALS patients showed an increase in NLRP3, ASC, caspase-1, and IL-1β, suggesting NLRP3 activation may be elevated in these patients.^229^ In addition, the SOD1 mutant mouse model of ALS exhibits an increased level of NLRP3 activation, as well as ASC speck formation and IL-1β maturation in its spinal cord.^229,230^ It was found that caspases-1 and IL-1 deficiencies restored the motor deficits associated with ALS in SOD1 mutant mice.^231^ Interestingly, one study found that NLRP3 was present in CD11b+ cells but not in Iba1+ cells in the spinal cord of SOD1 mice.^232^ This suggests that peripheral immune cells may also contribute to inflammation in neurodegenerative diseases. Furthermore, evidence also showed that NLRP3 inflammasome activation occurred in muscle and near neuromuscular junctions, which enhanced skeletal muscle degeneration in SOD1G93A mice.^233^ Altogether, there is still much to learn about the role of NLRP3 activation in ALS development.

MS is a common autoimmune disease in CNS characterized by oligodendrocyte attack and demyelination. It has been reported that MS patients have elevated caspase-1 and insulin-like growth factor 1 levels in their peripheral blood monocytes, brain tissues, and cerebrospinal fluid.^234,235^ In addition, a crucial function of NLRP3 in experimental autoimmune encephalomyelitis (EAE) is to prime CD4 + T cell migration through increasing the expression of chemotaxis-related protein, which indicates that the EAE animal model for MS involves the NLRP3 inflammasome.^236^ Neuromyelitis optica spectrum disorder (NMOSD) is another CNS autoimmune disease. MS and NMOSD share similar symptoms. Recent studies have identified that NLRP3 levels in the CSF were significantly increased in patients suffering from NMOSD or MS. In addition, NMOSD patients had higher CSF NLRP3 levels compared with MS patients.^237^ These findings indicate that levels of NLRP3 in CSF could be a potential diagnostic marker in NMOSD and MS.

Together, these findings support the notion that an increased susceptibility to aberrant inflammasome action and activity in various cell types of the CNS could make the brain more vulnerable to neurodegenerative changes, and subsequently promote the development of AD, PD, or other neurological diseases. There is a need for continued research in the development of drugs that target specific cell types or inflammasome signaling pathways. This research could provide valuable insights into how inflammasomes contribute to disease pathology in various neurodegenerative disorders. Such drugs may have significant therapeutic potential and should be further explored.

### Respiratory disorders

Inflammasomes are implicated in the pathogenesis of several respiratory disorders, including asthma, chronic obstructive pulmonary disease (COPD), pulmonary infection, pulmonary fibrosis, and acute respiratory distress syndrome (ARDS). As a result, inflammasomes have become a significant focus for research aimed at developing new diagnostic biomarkers and therapeutic avenues in pulmonary diseases and other respiratory disorders.

Asthma is a chronic inflammatory disease of the airways that affects inflammatory cells and cytokines in the lungs.^238^ Exposure to industrial products, microbes, or other allergens induces reversible limitations in airflow into and out of the lungs, as well as airway hyper-responsiveness. Persistent airway inflammation causes structural changes in airway tissues, known as airway remodeling, resulting in nonreversible airway obstruction and progressive loss of lung function. There is increasing evidence that NLRP3 inflammasome plays a role in asthma. Researchers found upregulated expression of *NLRP3* and of *IL‐1β* genes in phlegm obtained from the lungs of 127 asthmatic patients.^239^ The mechanism by which NLRP3 inflammasomes are activated in asthma is unclear. Chlamydia and Haemophilus infection, or OVA, titanium dioxide nanoparticles, and silica treatments stimulated the NLRP3, caspase-1, and IL‐1β expression, ultimately causing steroid-resistant neutrophil inflammation and hyperresponsiveness.^240^ Follistatin‐like 1 deficiency attenuates OVA‐induced mucus over secretion and airway mucin MUC5AC production, inhibits the NLRP3 and IL‐1β expression, inflammatory cytokines production, and inflammatory cell infiltration.^241,242^ Whether interventions on the NLPR3 inflammasome differ from other inflammation targets could serve as an additional avenue of research.

Activation of inflammasomes and alterations of their responses linked to the development of airway inflammation may also be seen in COPD.^243^ A comparison of COPD with smoking revealed elevated levels of NLRP3, Caspase-1, ASC, IL-1β, and IL-18 mRNA in peripheral blood mononuclear cells and bronchial tissues.^244^ However, the mRNA levels of NLRP3, Caspase-1, ASC, IL-1β, and IL-18 mRNA were higher in acute exacerbation of COPD (AECOPD) than those in COPD patients in the stable stage, suggesting a greater involvement of the NLRP3 inflammasome in AECOPD. Studies also found that cigarette smoke extract (CSE) stimulates the heat shock protein 60 expression and activates NLRP3 inflammasome through the TLR4-MyD88-NF-κB signal pathway.^245^ Smoking cessation is the most important COPD intervention for smokers. There is evidence that CSE induces pyroptosis via the ROS-NLRP3-caspase-1-GSDMD pathway in human bronchial epithelial cells.^246^ Particulate matter (PM2.5) also plays a role in lung injury: after PM exposure, Sirtuin1 (SIRT1) inhibits sterol regulatory element binding protein-1 (SREBP-1) and further decreases PIR and NLRP3 inflammasomes.^247^ In addition, the ROS-TRPM2-Ca2^+^-NLRP3 pathway also contributes to lung injury induced by PM 2.5.^248^ These studies indicate that the NLRP3 inflammasome can be activated via multiple pathways in lung injury, which provides a new therapeutic target for COPD.

Severe coronavirus disease 2019 (COVID-19) is a viral RNA infection that can cause persistent lung inflammation, dysregulation of cytokine production, sustained IFN response, as well as respiratory failure.^249^ Viruses could trigger the NLRP3 inflammasome. Postmortem study showed that patients with fatal COVID-19 were found to have abundant NLRP3, ASC, and caspase-1 in their lungs.^250^ As COVID-19 enters cells through the protein angiotensin-converting enzyme 2 (ACE2), dsRNA and ssRNA derived from the virus are recognized by TLR3 and TLR7, leading to elevated pro-IL-1β and pro-IL-18 levels, which are then cleaved into their mature forms once the NLRP3 inflammasome has been activated.^251^ NLRP3 can be directly activated by viral N protein.^252^ A correlation exists between levels of IL-1β, IL-18, lactate dehydrogenase, and COVID-19 severity in patients, which indicates that inflammasome activation and pyroptosis are involved in the pathology.^253,254^ COVID-19 patients have also consistently shown GSDMD in their serum.^255^ SARS-CoV-2 infected MISTRG6- human ACE2 (hACE2) humanized mouse model recapitulates the pathology, lung inflammation, dysregulation of cytokine production, sustained interferon response, as well as respiratory failure, with a human immune system.^256,257^ Infection and replication of SARS-CoV-2 in lung-resident macrophages play a crucial role in the development of the disease. When infected, human macrophages experience an inflammatory response that is controlled by CD16 and ACE2 receptors.^256^ This response includes the activation of inflammasomes, which leads to the release of IL-1 and IL-18, and pyroptosis. All of these factors contribute to the hyperinflammatory state of the lungs. However, inhibiting the NLRP3 inflammasome pathway can help reverse the chronic lung damage caused by this response. When inhibited with MCC950, the virus was released by infected macrophages, which indicated that the NLRP3 gene is activated to prevent SARS-CoV-2 infection.^258^ Together, early treatment targeting NLRP3 inflammasome could improve the prognosis of COVID-19.

Acute respiratory distress syndrome (ARDS) is an inflammatory disease that is characterized by diffuse alveolar injury hypoxemia, and acute respiratory failure.^259^ ARDS can lead to the development of pulmonary edema due to increased permeability of pulmonary microvascular endothelium, which impairs lung tissue ventilation. Caspase-1 and IL-18 promote ARDS development, and circulating IL-18 levels have been associated with disease severity and mortality.^260^ The recognition of LPS by TLR4 activates the NLRP3 inflammasome, as well as IL-1R1 expression on alveolar macrophage surfaces via the MyD88/NF-κB dependent pathway.^261^ LPS-TLR4 signals alveolar macrophages that increase ARDS by upregulating IL-1β-IL-1RI signaling. There is also evidence that NLRP3-mediated pyroptosis plays a role in ARDS pathogenesis. Extracellular histones induce alveolar macrophage pyroptosis via the NLRP3/caspase-1 pathway, which exacerbates lung inflammation in ARDS.^262^

The end result of inflammatory pulmonary diseases is fibrosis. The NLRP3 inflammasome mediates pulmonary fibrosis through the IL-1β-IL-1Rs-MyD88-NF-κB signaling pathway.^263^ Moreover, studies also found that NLRP3 inflammasome could transform lung endothelial cells into epithelial-mesenchymal transition, promoting pulmonary fibrosis. Caspase-1 and IL-1β play vital roles in pulmonary fibrogenesis. Caspases-1enzyme cleavages the pro-IL-1β and allows secretion of IL-1β.^264^ Elevated IL-1β has a profibrotic effect, and usually occurs in combination with higher expression of IL-1Rs in fibrogenesis.^265^ A study on mouse primary lung fibroblasts showed that the NLRP3 inflammasome increases IL-1β production, leading to lung fibrosis when induced by bleomycin.^266^ Collectively, these investigations have helped to clarify the role of inflammasomes in the development of pulmonary fibrosis and may lead to the discovery of new treatment targets for various respiratory disorders.

### Digestive disorders

Inflammasomes play a role in a variety of digestive disorders and have become a popular topic of research. Research focused on inflammasomes offers a better understanding of the mechanisms of several digestive diseases and conditions, including certain bacterial and viral infections, fatty liver disease (FLD), pancreatitis, and inflammatory bowel disease (IBD).

To date, the only harmful pathogenic bacterium that has been found to survive in human gastric mucosa is helicobacter pylori (HP). Some research suggests that inflammasome activation may be a contributing factor in the severity of HP infections. For example, one study found that NLRP3 and GSDMD levels were significantly higher in the gastric tissues of HP-infected individuals compared with healthy controls.^267^ In the innate immune cell neutrophils, inflammasome activation is stimulated by HP, which triggers K^+^ efflux and ROS production, resulting in an increase in IL-1β secretion.^268^ Notably, the elevated IL-1β levels were abolished in NLRP3-deficient neutrophils, suggesting that activation of the NLRP3 inflammasome plays an important role in the inflammatory response to HP. Additionally, NLRP3 knock-down or knock-out prevented gastritis in HP-infected mice.^269^ These findings suggest that HP bacteria may manipulate the machinery regulating the NLRP3 inflammasome to suppress the immune response.

Chronic infection with viral hepatitis is of high prevalence worldwide. Hepatitis B virus (HBV) is a viral infection that attacks the liver and can cause chronic hepatitis B (CHB).^270^ HBV-related acute-on-chronic liver failure patients have been shown to have higher levels of NLRP3, caspase-1, IL-1β, and IL-18 in their liver tissues.^271^ Moreover, the NLRP3, ASC, and IL-1β levels in liver tissues of CHB patients were positively correlated with the concentrations of HBV-DNA.^272^ This data suggests that long-term HBV infection activates the NLRP3 signaling pathway and promotes the IL-1β and IL-18-mediated injury of liver tissues. Similarly, patients with chronic hepatitis C have significantly increased serum IL-1β levels.^273^ Mechanistically, the Hepatitis C virus (HCV) RNA induces MyD88-mediated TLR7 signaling, activates the NLRP3 inflammasome pathway, and consequently triggers IL-1β production.^273^ Upon HCV infection, ASC binds to NLRP3, causing fragmentation of the Golgi.^274^ As a result, HCV replication increases and chronic liver inflammation occurs. Apart from antiviral agents, inhibiting the NLRP3 inflammasome and its associated cytokines could be a viable therapeutic approach to reduce liver inflammation.

FLD is a hepatic disease that results in the progressive buildup of fat in the liver. Patients with FLD have high serum caspase-1 levels, and these levels closely correlate with disease severity.^275^ Excessive fat accumulation in dead hepatocytes activates macrophages via NLRP3 and caspase-1.^276^ Researchers have shown that inhibiting pyroptosis through targeting NLRP3 inflammasomes can alleviate liver inflammation.^277,278^ NLRP3 inflammasome is implicated in the development of FLD in mice, and diet-induced steatohepatitis is prevented in mice lacking NLRP3 inflammasome function.^279^ The inhibition of the NLRP3 inflammasome significantly reduced inflammation, lipid accumulation, and fibrosis in FLD.^280,281^ In contrast, there is evidence that NLRP3 deficiency plays a harmful role via increasing serum alanine transaminase (ALT) and aspartate transaminase (AST) levels. FLD model mice lacking NLRP3 displayed a higher triglyceride content, liver injury scores, and adipose tissue inflammation.^282^ More research is necessary to explain the contradictory results mentioned above. Long-term heavy drinking can cause alcoholic liver disease, and patients with severe liver damage had higher mRNA levels of NLRP3, IL-1β, IL-18, and caspase-1 compared to patients with milder liver damage.^283^ Long-term alcohol intake facilitated liver damage and promoted NLRP3, ASC, caspase-1, and IL-1β expression in wild-type mice.^284^ Chronic alcohol consumption can cause metabolic disorders characterized by excess production of uric acid and ATP, which can trigger NLRP3 inflammasome activation and mitochondrial damage.^285^ Aryl hydrocarbon receptor downregulation and activation of TXNIP are the primary mechanisms responsible for ethanol-induced NLRP3 activation in human macrophages.^286^ Taken together, these studies suggest that interventions aimed at inhibiting NLRP3 inflammasome activation could help abate alcoholic liver disease.

Pancreatitis is an inflammatory condition that can be broken down into three main types: acute pancreatitis, severe acute pancreatitis, and chronic pancreatitis. NLRP3 plays a crucial role in pancreatic tissue inflammation. NLRP3, caspase-1, pro-IL-1β, and pro-IL-18 activation were observed in a mouse model of acute pancreatitis.^287^ Additionally, Inhibition of P2X7R could reduce chronic pancreatic inflammation and fibrosis by attenuating NLRP3-mediated IL-1β and IL-18 secretions in a mouse model of chronic pancreatitis.^288^ The NLRP3 inflammasome is also believed to contribute to the development of fibrosis in patients with chronic pancreatitis. It activates pancreatic stellate cells (PSCs), which release a large amount of extracellular matrix (ECM).^289^ By inhibiting NLRP3, PSC, activation and ECM deposition can be reduced, ultimately relieving pancreatic fibrosis.^290^ Pancreatic cells recognize PAMPs and DAMPs in tissue damaged by acute pancreatitis, triggering NF-κB and NLRP3 inflammasome expression, caspase-1 maturation,^291^ and pyroptosis.^287^ In mice with severe acute pancreatitis, NLRP3 deficiency was also found to alleviate inflammatory complications.^292^ These studies suggest that inflammasome-targeted treatments could help alleviate or even treat many cases of pancreatitis.

Inflammatory bowel disease (IBD) is a chronic, recurring gastrointestinal disorder in which no structural or biochemical abnormalities occur. Ulcerative colitis (UC), Crohn’s disease (CD), infectious agents, and environmental factors are risk factors for IBD.^293^ UC and CD patients had increased levels of NLRP3 and IL-1β.^294^ Moreover, NLRP3 inflammasome and IL-1β levels are increased in IBD patients’ mucosa, and these levels closely correlate with disease severity.^295^ The development of intestinal inflammation was impaired by the deletion and inhibition of NLRP3, which indicates that IBD is promoted by overactivation of the NLRP3 inflammasome.^296^ In contrast, studies have demonstrated that the NLRP3 inflammasome regulates mucosal immune responses and intestinal homeostasis.^297^ Proliferation of intestinal endothelial cells requires NLRP3-induced IL-18.^298^ NLRP3 inflammasome activation-induced IL-1β and IL-18 protect against colitis and colitis-associated tumorigenesis in mice.^299^ Whether the NLRP3 inflammasome contributes to IBD in a beneficial or pathogenic way is a topic of debate. The inconsistent results may stem from variations in experimental protocols, the strains of mice utilized, or dose-response effects in microbiota. Additionally, certain genetic mutations in the NLRP3 inflammasome also appear to play a role in the pathology of IBD. NLRP3, encoded by RS772009059 (R779C), in 3 patients showed early-onset IBD.^56^ A positive correlation was also found between the incidence of severe diseases and NLRP3 inflammasome activity in macrophages when R779C is present in DSS-induced acute colitis models.^56^ More studies on how the NLRP3 inflammasome regulates inflammation will inspire new approaches to therapeutics for IBD.

### Urogenital disorders

Inflammasomes have been implicated in the pathology of urogenital disorders, including renal, cystic, prostate, and ovarian diseases. Acute kidney injury (AKI) is characterized by a sudden decrease in glomerular filtration rate and an increase in waste product accumulation, which can lead to ischemia-reperfusion injury (IRI). NLRP3, IL-1β, and IL-18 levels are elevated in IRI.^300^ The NLRP3 inflammasome plays a vital role in AKI. NLRP3 inflammasome activation and mitochondrial damage were detected in IR-induced AKI model mice, and damaged mitochondria further activate the NLRP3 inflammasome via the mROS-TXNIP-NLRP3 pathway.^301^ NLRP3 deletion protected the kidney from further inflammatory damage and injury.^302^ Moreover, pyroptosis also occurred in tubular epithelial cells of renal IRI mice.^303^ Another study discovered that protection from IRI was observed in NLRP3 knockout mice, but not in ASC knockout or caspase-1 knockout mice. This suggests that NLRP3 may directly impact renal IRI’s tubular epithelial cells.^304^ Additionally, P2X7R deficiency attenuates NLRP3 inflammasome formation and kidney injury.^305^ Cathelicidin-related antimicrobial peptide (CRAMP) deficiency promotes inflammatory responses and apoptosis via NLRP3 inflammasome overexpression.^306^ It indicates that CRAMP plays a protective role in the kidney by inhibiting the NLRP3 inflammasome activation. Additionally, NLRP6 also has been implicated in AKI. NLRP6 expression is downregulated when nephrotoxic kidney injury occurs.^307^ NLRP6 deficiency is believed to exacerbate the severity of AKI by inhibiting the phosphorylation of ERK1/2 and p38 MAPK, and suppressing the nephroprotective gene Klotho expression.^308^ NLRC4 expression is increased after IRI. Treatment with RMT3-23, a neutralizing antibody against T cell immunoglobulin domain and mucin domain-containing molecule-3 (Tim-3), decreases NLRC4 expression in IRI.^309^ This study suggests that Tim-3 mediates NLRC4 inflammasome activation in AKI. It has been observed that the expression of NLRC5 is elevated in mice that have undergone IRI.^310^ NLRC5 deficiency suppresses oxidative stress and apoptosis by promoting the PIK3/Akt signaling pathway in HK-2 cells.^310^ Mechanistically, NLRC5 downregulates ERK1/2 and Akt signaling, exacerbating the inflammatory response and apoptosis in tubular epithelial cells. Therefore, these findings present a novel perspective on therapeutic implications in AKI patients.

The progression of renal disease is accompanied by tubulointerstitial inflammation and fibrosis. Evidence shows that NLRP3 plays a key role in the progression of unilateral ureteral obstruction nephropathy via the NLRP3 inflammasome pathway.^311^ NLRP3 deficiency suppresses tubular injury, tubulointerstitial inflammation, and fibrosis in NLRP3-knockout mice, and these observations are in line with the inhibition of caspase-1 activity as well as IL-1β/IL-18 production.^312^ In contrast, it was also reported that NLRP3 has a protective effect on early tubular injury. NLRP3 mRNA levels are elevated in renal tubular epithelial cells, attenuating renal injury by preserving renal integrity.^313^ These controversial results indicate that NLRP3 functions distinctly at the different stages of obstruction nephropathy. NLRP3 is targeted by drugs like aliskiren, fluorofenidone, and mefunidone, which decrease NLRP3 inflammasome activity and suppress the release of IL-1β in obstruction nephropathy.^314^

Inflammation facilitates systemic lupus erythematosus-induced lupus nephritis. NLRP3 inflammasome is found in podocytes and contributes to cellular injury and proteinuria during lupus nephritis development.^315^ NLRP3 inflammasome-mediated IL-1β and IL-18 production have been shown to be elevated in the renal tissue and podocytes of mice with renal impairments.^316^ Mechanistically, NLRP3 inflammasome activation is regulated by RIP3 in podocytes.^317^ In addition, activation of glycogen synthase kinase 3β and P2X7R activate the NLRP3/IL-1β pathway and subsequently aggravate the development and progression of lupus nephritis.^318^ The cancer treatment drug tris dipalladium could inhibit MAPK (ERK, JNK)-mediated NLRP3 activation and the autophagy/NLRP3 pathway, alleviating tubulointerstitial inflammation and restoring renal function.^319^ These data suggest that the NLRP3 inflammasome plays an important role in lupus nephritis pathophysiology.

IgA nephropathy is a chronic, progressive glomerulonephritis characterized by the deposition of the IgA immune complex in the glomerular mesangium. NLRP3 levels are significantly upregulated in patients with IgA nephropathy, as well as in the IgA nephropathy mouse model.^320,321^ The IgA immune complex activates the NLRP3 inflammasome, leading to mitochondrial dysfunction and mROS overproduction in macrophages. NLRP3 deficiency attenuates renal injury in IgA nephropathy.^322^ Furthermore, there is evidence that IgA induces podocyte NLRP3 expression as well as macrophage trans-differentiation, which leads to renal fibrosis in IgA nephropathy.^320^ Intriguingly, patients with IgA nephropathy have a worse prognosis when their NLRP3 mRNA expression is low.^323^ Reduced levels of NLRP3 mRNA and protein in the tubules may reflect a loss of the tubular epithelial phenotype and cell death. Further investigation needs to be done to clarify the exact role of NLRP3 inflammasome in IgA nephropathy.

The NLRP3 inflammasome and IL-1β release have been implicated in the development of urinary tract infections. CFT073, a strain of uropathogenic *Escherichia coli* (UPEC), increased caspase-1 activity and promoted IL-1β release from bladder epithelial cells.^324^ The increase in IL-1β release was initially suppressed and later induced by the biphasic effect of α-hemolysin. This was mediated by α-hemolysin through NLRP3 inflammasome activation in an NF-κB-independent manner. These findings suggest that α-hemolysin can modulate the NLRP3 inflammasome activity in bladder epithelial cells. Urine contains many substances that can be potentially harmful if not eliminated, and this is particularly evident with bladder stones. Bladder stones are hardened mineral clumps that are composed of calcium pyrophosphate (CPPD) and monosodium urate (MSU). It has been found that CPPD and MUS can trigger caspase-1 in urothelial cells. However, the activation of caspase-1 can be reduced by NAC and Verapamil, which are ROS scavengers and recognized as down-regulators of TXNIP.^325^ This data suggests that the CPPD and MSU cause NLRP3 inflammasome activation in bladder urothelium, and this mechanism is dependent on ROS generation and TXNIP expression.

The NLRP3 inflammasome has also been implicated in bladder outlet obstruction. Any number of conditions, such as bladder stones, can cause bladder outlet obstruction. During bladder outlet obstruction in the urothelium, NLRP3 is activated, eventually leading to fibrosis and decompensation. However, an NLRP3 inhibitor called Glyburide can block the inflammation and prevent dysfunction of bladder outlet obstruction in its early stages.^326^ This suggests that the NLRP3 inflammasome plays a critical role in bladder outlet obstruction. A blocked bladder outlet stimulates fibrosis, which is responsible for chronic bladder decompensation in this condition. Research has demonstrated that IL-1β receptor antagonists can prevent collagen production in the bladder caused by bladder outlet obstruction, indicating the involvement of the NLRP3/IL-1β pathway in fibrosis.^327^ Additionally, IL-1β was found to stimulate collagen expression in isolated urothelial cells. These data suggested that NLRP3-mediated IL-1β release triggers fibrosis during bladder outlet obstruction by driving collagen production in urothelial cells.

Prostatitis is an inflammatory disease which has both infectious and non-infectious causes. In a study using rats, it was discovered that a hormone imbalance can cause chronic non-bacterial prostatitis.^328^ This condition leads to an increase in expression levels of NLRP3, ASC, and Caspase-1, causing inflammasome activation and inflammatory responses. However, melatonin was found to be effective in suppressing prostate inflammation and pelvic pain, by inhibiting the NLRP3 inflammasome signaling pathway. Additionally, the activation of Sirt1 was observed in an experimental autoimmune prostatitis mouse model, further demonstrating the potential therapeutic benefits of melatonin. In another study, *Trichomonas vaginalis* stimulation was shown to increase the expression of NLRP3, ASC, Caspase-1, and IL-1β, while inhibition of NLRP3 and Caspase-1 decreased the *T. vaginalis*-induced IL-1β secretion in a prostate epithelial cell line (RWPE-1).^329^ These studies provide evidence that the NLRP3 inflammasome is associated with the development of prostatitis.

Polycystic ovary syndrome is a type of infertility mainly caused by hyperandrogenism. In polycystic ovary syndrome models, ovarian TLR4 expression, as well as serum anti-Müllerian hormone, testosterone, caspase1, IL-1β, and insulin levels were increased.^330–332^ Mechanistically, activation of NLRP3 in upregulated the 3β-hydroxysteroid dehydrogenase and androgen receptor (AR) expression and downregulated the follicle-stimulating hormone receptor expression, inhibition of NLRP3 suppressed the expression of ASC, GSDMD-C, and AR.^331^ These results indicate that activating the NLRP3 inflammasome is crucial for the progression of hyperandrogen-induced polycystic ovary syndrome.

The NLRP3 inflammasome plays a role in ovarian aging and female fertility. NLRP3, caspase-1, and IL-1β expression were increased in granulosa cells from mice with ovarian insufficiency. Additionally, NLRP3 expression increases in the ovary as wild-type mice age.^333^ Ablation of NLRP3 leads to improved pregnancy and survival rates, as well as hormone levels in the ovaries of these mice.^333^ These findings suggest that elevated NLRP3 inflammasome may contribute to age-related deficits in female fertility. Additionally, endometriosis is an estrogen-dependent chronic inflammatory syndrome. It can lead to infertility, and hormone-based treatments are usually used to treat it. Research suggests that the NLRP3 inflammasome could be involved in the development of endometriosis. Studies have shown that NLRP3 levels are higher in ovarian endometriosis samples, and using MCC950 to inhibit NLRP3 has been shown to decrease IL-1β concentrations in cyst-derived stromal cells.^334^ The results suggested that NLRP3/IL-1β is crucial for the pathogenesis of endometriosis.

### Blood and lymphatic system disorders

A growing number of researchers are exploring the effect that aberrant inflammasome activation has on disorders of the blood and lymphatic system. To date, several studies suggest that inflammasomes play a role in the pathogenesis of leukemia, myeloproliferative neoplasms, myelodysplastic syndrome (MDS), lymphoma, and autoimmune diseases.

Leukemia refers to a group of clonal hematological diseases that affect the maturation and proliferation of myeloid cells and lymphocytes. Leukemia has both acute and chronic forms, including acute myeloid leukemia (AML), acute lymphocytic leukemia (ALL), chronic lymphocytic leukemia (CLL), and chronic myeloid leukemia (CML). Studies have reported that dysregulated IL-1β secretion positively correlates with disease progression and poor prognosis in leukemia.^335^ Research has identified the KrasG12D mutation as a genetic risk factor for Leukemia. It has been observed that this mutation can activate the NLRP3 inflammasome, resulting in myeloproliferation and cytopenia. Nevertheless, experiments with KrasG12D murine models have demonstrated that NLRP3 deficiency can reverse these effects.^336^ Patients newly diagnosed with AML have also been found to have increased NLRP3 expression in their bone marrow mononuclear cells and their peripheral blood mononuclear cells (PBMCs).^337^ Glucocorticoids are often used to treat patients with ALL. NLRP3 and caspase-1 are significantly higher in ALL cells resistant to glucocorticoids, as caspase-1 cleaves the glucocorticoid receptor.^338^ In contrast, NLRP3 expression was significantly lower in CLL lymphocytes than in healthy donors, whereas P2X7R expression was higher.^339^ Aside from activating NLRP3 inflammasomes, P2X7R also inhibits apoptosis and promotes cell proliferation.^339^ NLRP3 downmodulation triggers P2X7R expression, which consequently leads to tumor growth. In addition, curcumin could induce the expression of AIM2, NLRC4, and IFI16 inflammasomes in leukemia cells U937, which subsequently activated caspase 1, promoted GSDMD cleavage, as well as induced pyroptosis.^340^ Altogether, targeting the inflammasomes could have therapeutic effects on leukemia.

Myeloproliferative neoplasms include polycythemia vera, essential thrombocythemia, and primary myelofibrosis. Analysis of patients showed that the IL-1β levels were increased in all three different types of myeloproliferative neoplasms.^341^ Moreover, JAK2V617F positive macrophages produced greater IL-1β and IL-18, which promoted the production and activation of neutrophils and the entry of leukocytes into lesion.^342^ Additionally, AIM2, IL-1β, and caspase-1 were significantly increased in JAK2V617F positive cells.^343^ This data indicates that inflammasomes are vital in the pathogenesis of myeloproliferative neoplasms. MDS describes a group of malignant preleukemic HSC malignancies resulting from abnormal and ineffective hematopoiesis. The activation of the NLRP3 inflammasome was suggested as contributing to MDS. The alarmin S100A9 could induce ROS generation, which subsequently activates the NLRP3 inflammasome, leading to IL-1β secretion and pyroptosis.^344^

It has been reported that inflammasomes also play a role in lymphoma development. The NLRP3 inflammasomes are implicated in numerous cancers as a pro-tumorigenic factor. Patients with diffuse large B cell lymphoma (DLBCL) exhibit immunosuppression. This is characterized by significantly increased levels of IL-18 in lymphoma tissues, which is positively correlated with the expression of programmed death ligand 1 (PD-L1).^345,346^ PD-L1 is induced by NLRP3 inflammasome activation, and this reduces the proportion of cytotoxic T cells in DLBCL cell lines. However, in vivo blockade of the NLRP3 inflammasome inhibits lymphoma growth and suppresses anti-tumor immunity. This is achieved by decreasing the expression of PD-L1 in the tumor microenvironment and downregulating the proportion of PD-1/TIM-3-expressing T cells, regulatory T cells, myeloid-derived suppressor cells, and tumor-associated macrophages. It has been shown that the NLRP3 inflammasome regulates PD-L1 and immune cells to promote immunosuppression, while IL-18 negatively impacts anti-lymphoma immunity in vivo. Patients with Sjögren’s syndrome (SS) have hyperfunctioning P2X7R, which triggers acute inflammatory responses through the NLRP3 inflammasome.^347^ SS patients may develop mucosa-associated lymphoid tissue non-Hodgkin’s lymphoma (MALT-NHL). P2X7R, NLRP3, caspase-1, and IL-18 expression were higher in patients with germinative centers and autoantibody-positive individuals and were significantly higher in patients who developed a MALT-NHL during follow-up.^347^ Primary cutaneous T cell lymphomas most commonly occur as mycosis fungoides (MF), NLRP1 expression was increased in the early stages of MF, whereas caspase 1, IL-1β, and IL-18 levels were increased during both the early and late stages of MF formation.^348^ Intriguingly, in cutaneous T cell lymphoma (CTCL), unassembled NLRP3 can translocate to the malignant CD4 + T cells nucleus, and bind to human IL-4 promoter to regulate the IL-4 expression, IL-4 can inhibit the NLRP3 inflammasome assembly.^349^ It has been discovered that infections caused by Kaposi’s sarcoma-associated herpesvirus (KSHV) in B and endothelial cells are linked to the development of Kaposi’s sarcoma and primary effusion B-cell lymphomas, respectively. During primary infection of KSHV in endothelial cells, B cells in Kaposi’s sarcoma endothelial and primary effusion B-cell lymphomas can secrete IL-1β and IL-18 upon caspase-1 activation. It has been found that IFI16 interacts with ASC, but not AIM2 or NLRP3, and subsequently procaspase-1, to form an effective inflammasome in KHSV.^350^ Another study reported that mutations in IL-18 (rs1946518) and NFκB-94 ins/del (rs28362491) promoted the susceptibility to B-cell NHL.^345,351^ Additionally, allele G in IL-18 was shown to be remarkably associated with the risk of lymphoma.^345^ These findings show that specific inflammasomes play different roles in lymphoma development.

Patients with systemic lupus erythematosus (SLE) suffer from disordered Th1/Th2 and Treg/Th17 balances, evidence has demonstrated that NLRP3, NLRP1, NLRC4, and AIM2 were involved in the regulation of Th1, Tfh, and Th17 cell-mediated immune responses. Leptin contributes to SLE development by activating the NLRP3 inflammasome and promoting Th17 cell differentiation in lupus erythematosus mice.^352^ SLE is identified by the presence of anti-dsDNA antibodies, which can be measured using an antinuclear (ANA) test. Additionally, in human monocytes, anti-dsDNA antibodies can activate the NLRP3 inflammasome and cause the secretion of IL-1β, further contributing to the pathogenesis of SLE.^353,354^ Research has shown that AIM2 can encourage the production of B cells in patients with SLE.^355^ People with SLE tend to have higher levels of AIM2 mRNA in their liver, PBMCs, and spleen than healthy individuals. AIM2 has also been found to help prevent SLE-inhibiting DNA-induced IFN signals.^356^ However, some studies suggest that AIM2 may contribute to the development of Th17 cells in SLE. In mice, inhibiting AIM2 expression has been found to greatly improve SLE symptoms.^355,357^ While there is research on the roles of NLRP3 and AIM2 in SLE, the functions of other inflammasomes in the disease are not yet fully understood.

Rheumatoid Arthritis (RA) is an autoimmune disease characterized by immune dysregulation and joint inflammation. Patients with RA have elevated levels of NLRP3 and IL-1β secretion in their peripheral blood mononuclear cells (PBMC),^358^ as well as an increase in IL-18 in their bronchoalveolar lavage fluid (BALF) if they have RA-recurrent interstitial pneumonia (RA-UIP).^359^ NLRP3 deficiency has been found to inhibit Th17 cell differentiation in RA patients, indicating that NLRP3 promotes the differentiation of Th17 cells, which can exacerbate inflammation.^360^ Mechanistically, calcium-sensitive receptors (CaSR) in RA patients can activate the NLRP3 inflammasome, releasing IL-1 and promoting joint swelling.^361^ PTX3 and complement C1q promote NLRP3 inflammasome hyperactivation and scorching in RA patients.^362^ Additionally, researchers are increasingly studying AIM2 inflammasomes as cytoplasmic receptors in RA. It was observed that the levels of serum AIM2 were lower in RA patients compared to healthy controls; however, ASC, caspase-1, and IL-1β levels were higher.^363^ Individuals diagnosed with RA have elevated levels of mDNA in their plasma and synovial tissue compared to healthy individuals.^364^ They also have a higher chance of activating AIM2 inflammasomes. In addition, the adjuvant arthritis rat model showed activation of the NLRP1 inflammasome.^365^ Monocytes from RA patients were found to have an increased expression of NLRC4.^366^ However, more research is needed to determine the exact role of inflammasomes and their association with the susceptibility and severity of RA.

The use of inflammasome-targeting therapy has emerged as a possible therapeutic strategy for blood and lymphatic system disorders. The associations between blood and lymphatic system disorders and the inflammasomes are active research areas. It is imperative to conduct further research to fully understand the role of inflammasomes in these disorders and to develop effective treatments for them.

### Other disorders

In addition to the disease mentioned above, other disorders are associated with abnormal inflammasome activity, including gout, diabetes, and obesity. Gout is a common disorder characterized by the MSU crystals depositing in the articular and non-articular structures. MSU crystals are a typical DAMP that induced the NLRP3 inflammasome activation, and the IL-1β release is vital in the initiation of inflammatory gout attacks or flares.^367^ MSU stimulation resulted in decreased knee neutrophil infiltration in mice lacking critical components of inflammasomes (NLRP3, ASC, or caspase-1).^368^ An endogenous DAMP, cold-inducible RNA-binding protein, activates MSU-stimulated neutrophil infiltration via an NLRP3/ASC/caspase-1/IL-1β/MyD88 pathway-mediated CXC-motif receptor 2-dependent process.^369^

Diabetes type 2 (T2D) is a chronic inflammatory disease characterized by insulin resistance, upregulated circulating TNF, interleukin, and adipokines levels. IL-1β hinders insulin sensitivity by phosphorylating insulin receptor substrate-1, leading to the insulin-induced PI3K-Akt signaling disruption in insulin-targeted cells.^370^ Studies have shown that individuals with T2D have higher levels of NLRP3 inflammasome activity in their myeloid cells than those without the condition.^371^ Research conducted on mice without NLRP3, ASC, or caspase-1 genes has revealed that they exhibit better glucose tolerance and insulin sensitivity when consuming high-fat diets.^372,373^ A 37-amino-acid peptide hormone, islet amyloid polypeptide, released by β-cells along with insulin, can form an amyloid structure in the pancreatic islets of T2D patients.^374^ It has been found that endocannabinoids can trigger the production of IL-1β through the NLRP3 inflammasome via the peripheral CB1 receptor, which can result in the death of pancreatic β-cells.^375^ In rat islets treated with anandamide, an endocannabinoid, levels of ASC and caspase-1 activation were increased, and IL-1β secretion was upregulated in mouse macrophage cell line RAW264 in an NLRP3-dependent manner. In addition, it has been found that saturated fatty acids can also trigger T2D by activating the NLRP3 inflammasome.^376^ The NLRP3 inflammasome can sense ceramide, which is a type of saturated fatty acid, leading to the activation of caspase-1 in mouse bone marrow-derived macrophages and epididymal adipose tissue explants.^377^ On the other hand, unsaturated fatty acids have been shown to enhance insulin sensitivity by decreasing the production of IL-1β.^276^

Obesity is an adipocyte hypertrophy characterized in part by immune cell infiltration-induced adipose tissue expansion. Obesity can increase multiple metabolic disorders. In obese patients, NLRP3 and ASC/PYCARD expression is increased.^372^ Research has shown that the inhibition of IL-1β, but not IL-18, can enhance adipogenic gene expression. This indicates that caspase-1 plays a role in regulating adipogenesis through IL-1β.^378^ Studies have also revealed that caspase-1 is essential for the formation of adipose tissue. In mice that lacked caspase-1, there was a decrease in adipocyte size, a reduction in fat mass, an increase in the expression of adipogenic genes, and an improvement in insulin sensitivity.^379^ In addition, NLRP3-, ASC-, and caspase-1-deficient mice were found to be protected against obesity induced by a high-fat diet.^380^ Researchers also have shown that mice lacking IL-18 developed obesity due to increased food intake.^381^ Further studies are needed to clarify the mechanism and accurate role of the inflammasomes and caspase-1 activation in adipocytes and the pathogenesis of obesity.

## Inflammasome-targeted therapy

**Fig. 5:**
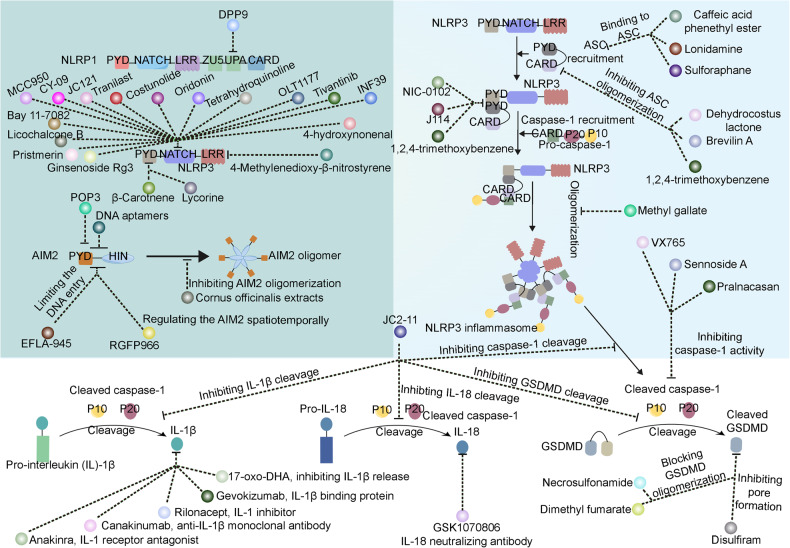
Inhibitors of the inflammasomes and inflammasome-related pathways. Distinct inhibitors target NLRP1, NLRP3, AIM2, ASC, caspase-1, interleukin-1β, interleukin-18, or GSDMD to inhibit the inflammasome activation and subsequent pyroptosis and cytokine release. The figure was created with the assistance of FIGDRAW

Different inflammasomes are increasingly recognized in various diseases, thus targeting inflammasome signaling pathways has the potential to develop new strategies for therapeutic intervention (Fig. 5). Currently, the FDA-approved drugs primarily target inflammasome-related pathways rather than directly targeting inflammasomes themselves (Table 1). Thus, more researches, however, are being conducted in the development and evaluation of inflammasome-targeting therapies (Table 2).

**Table 1:** FDA approved inflammasome-related drugs and their applications

Drug name	Target	Year	Initial applications	Recent applications	Most common adverse reactions	BLA
Anakinra	IL-1	2001	RA	CAPS, DIRA	Injection site reaction, worsening of rheumatoid arthritis, upper respiratory tract infection, headache, nausea, diarrhea, sinusitis, arthralgia, flu like-symptoms, and abdominal pain (incidence ≥ 5%)	103950
Rilonacept	IL-1	2008	CAPS	FCAS, MWS	Injection-site reactions and upper respiratory tract infections	125249
Canakinumab	IL-1β	2009	CAPS	FCAS, MWS	Sopharyngitis, diarrhea, influenza, headache, and nausea	125319

*RA* rheumatoid arthritis, *CAPS* cryopyrin-associated periodic syndromes, *DIRA* deficiency of interleukin-1 receptor antagonist, *FCAS* Familial Cold Autoinflammatory Syndrome, *MWS* Muckle-Wells Syndrome

**Table 2:** Clinical trials of inflammasome pathway related drugs

NCT number	Drug name	Target	Conditions	Study Type and/or phase	Enrollment	Arms	Study completion date
NCT05658575	OLT1177	NLRP3	Acute gout fare, gout attack, gout flare, gouty arthritis, gout arthritis, joint pain	Interventional, Phase2/3	300	A: Dapansutrile (also called OLT1177) B: Placebo Tablet	2023–10
NCT04540120	OLT1177	NLRP3	Covid19, cytokine release syndrome	Interventional, Phase2	49	A: OLT1177 Capsules B: Placebo Capsules	2022–07
NCT03595371	OLT1177	NLRP3	Schnitzler syndrome	Interventional, Phase2	10	A: OLT1177 Capsules	2023–02
NCT02104050	OLT1177	NLRP3	Osteoarthritis, pain	Interventional, Phase 2	202	A: OLT1177 gel B: Placebo gel	2015–08
NCT01768975	OLT1177	NLRP3	Osteoarthritis of the Knee	Interventional, Phase 2	79	A: OLT1177 gel B: Placebo gel	2013–08
NCT03534297	OLT1177	NLRP3	Systolic Heart Failure	Interventional, Phase 1	30	A: OLT1177 Capsules B: Placebo Capsules	2019–11
NCT02134964	OLT1177	NLRP3	Healthy	Interventional, Phase 1	35	A: OLT1177 Capsules B: Placebo Capsules	2014–12
NCT01636141	OLT1177	NLRP3	Healthy	Interventional, Phase 1	36	A: OLT1177 gel B: Placebo gel	2012–08
NCT05130892	Colchicine, tranilast, and oridonin	NLRP3	NLRP3, high-sensitivity C-reactive protein, percutaneous coronary intervention	Interventional, Phase 4	132	A: Colchicine group B: Tranilast group C: Oridonin group D: Non-intervention group	2023–02
NCT05855746	Colchicine	NLRP3	Acute Myocarditis	Interventional, Phase 3	300	A: Colchicine B: Placebo	2027–06
NCT05734612	Colchicine	NLRP3	Reperfusion injury, myocardial	Interventional, Phase 3	80	A: Colchicine B: Placebo	2023–03
NCT04322565	Colchicine	NLRP3	Coronavirus infections, viral pneumonia	Interventional, Phase 2	193	A: Colchicine B: Standard of care	2021–10

NCT04867226	Colchicine	NLRP3	Coronavirus infection	Interventional, Phase 2	100	A: Colchicine drug B: Usual care	2021–06
NCT05118737	Colchicine	NLRP3	COVID-19 pneumonia	Interventional, early Phase 1	230	A: Colchicine B: Control	2022–08
NCT03923140	Tranilast	NLRP3	Cryopyrin-Associated Periodic Syndromes	Interventional, Phase 2	71	A: Tranilast	2024–10
NCT01109121	Tranilast	NLRP3	Moderate to severe gout, hyperuricemia	Interventional, Phase 2	112	A: Allopurinol 400 mg QD B: Tranilast 300 mg QD + Allopurinol 400 mg QD C: Tranilast 300 mg QD + Allopurinol 600 mg QD	2011–01
NCT04047095	β-Carotene	NLRP3	Cardiac Surgery	Interventional	55	A: normal meal plus one sachet immune nutrients at 8 a.m., 1 p.m. and 6 p.m. B: normal meal at 8 a.m., 1 p.m. and 6 p.m.	2021–10
NCT03005496	β-Carotene	NLRP3	PreTerm birth	Interventional, Phase 4	56	A: Nifedipin + Dexamethasone + Zinc + β-Carotene + Vitamin D3 B: Nifedipin + Dexamethasone	2017–06
NCT03842709	Pramipexole	NLRP3	Chronic Pain	Interventional, early Phase 1	45	A: Pramipexole Oral Tablet B: Placebo	2021–02
NCT02375685	Gevokizumab	IL-1β	Chronic Uveitis	Interventional, early Phase 3	71	A: Gevokizumab	2015–11
NCT01965145	Gevokizumab	IL-1β	Behcet’s Uveitis	Interventional, Phase 3	84	A: Gevokizumab B: Placebo	2015–09
NCT01835132	Gevokizumab	IL-1β	Scleritis	Interventional, Phase 1/2	8	A: Gevokizumab	2016–02
NCT01211977	Gevokizumab	IL-1β	Muckle Wells Syndrome, autoinflammatory, Behcet’s Disease	Interventional, Phase 1/2	21	Not provided	2011–04

NCT02723786	GSK1070806	IL-18	Kidney transplantation	Interventional, Phase 2	7	A: GSK1070806 3 mg/kg IV	2018–03
NCT01648153	GSK1070806	IL-18	Diabetes Mellitus	Interventional, Phase 2	37	A: GSK1070806 0.25 mg/kg B: GSK1070806 5 mg/kg C: Placebo (Saline)	2014–01
NCT03522662	GSK1070806	IL-18	Behcet’s Disease	Interventional, Phase 2	12	A: GSK1070806	2020–04
NCT05590338	GSK1070806	IL-18	Dermatitis, atopic	Interventional, Phase 1	38	A: GSK1070806, intravenous (IV) infusion B: GSK1070806, IV bolus C: Placebo	2023–12
NCT01035645	GSK1070806	IL-18	Inflammatory bowel diseases	Interventional, Phase 1	78	A: GSK1070806 B: Placebo	2012–07
NCT04485130	Disulfiram	IL-18	Covid19	Interventional, Phase 2	11	A: Disulfiram B: Placebo	2022–02
NCT02561481	Sulforaphane	NLRP3 inflammasome activation pathway	Autism spectrum disorder	Interventional, Phase 1/2	60	A: Sulforaphane B: Placebo	2020–01
NCT04972188	ZYIL1	NLRP3 inflammasome pathway	Healthy	Interventional, Phase 1	18	A: ZYIL1 Capsule	2021–10
NCT04731324	ZYIL1	NLRP3 inflammasome pathway	Healthy	Interventional, Phase 1	30	A: ZYIL1 Capsule	2021–06
NCT04409522	Melatonin	NLRP3 inflammasome pathway	COVID-19	Interventional	55	A: Melatonin B: The usual treatment	2020–09
NCT05567068	Atorvastatin	mTOR/NLRP3 inflammasome pathway	Inflammatory Bowel Diseases	Interventional, Phase 2	44	A: Atorvastatin 80 mg B: Mesalamine	2027–09
NCT05781698	Fenofibrate	mTOR/NLRP3 inflammasome pathway	Inflammatory Bowel Diseases	Interventional, Phase 2	60	A: Mesalamine B: Mesalamine + Fenofibrate	2024–06
NCT 05276895	Glycyrrhizin	NLRP3 inflammasome activation and NF-κB signaling pathway	Osteoarthritis	Interventional	60	A: Quercetin + Fisetin B: Quercetin + Fisetin + Glycyrrhizin C: Placebo	2024–12

### Sensor protein modulation

In recent years, there has been significant interest in developing inhibitors of inflammasome sensor proteins as potential therapeutics for inflammatory diseases. Cytosolic DPP9 binds to NLRP1 C terminus and inhibits inflammasome activation.^382,383^ Recent studies have revealed that the NLRP3 inhibitor MCC950 can effectively inhibit the activation of NLRP3 by directly targeting its NACHT domain.^384^ Specifically, MCC950 has been shown to block the Walker B motif within the NACHT domain of NLRP3, thus preventing ATP hydrolysis by NLRP3. Tranilast is a drug used for allergies that can prevent NLRP3 assembly by binding directly to its NACHT domain and preventing it from forming. Doing this also stops direct interactions between NLRP3 molecules and disrupts their natural interaction with ASC.^385^ Another drug, CY-09, interacts with the NLRP3 Walker A motif and removes ATP bound to NLRP3 without affecting NLRP1 or NLRC4.^386^ N-benzyl 5-(4-sulfamoylbenzylidene-2-thioxothiazolidin-4-one analogs), which are hybrids of CY-09, selectively inhibit the formation of oligomer specks of NLRP3 and ASC. This reduces the assembly of the NLRP3 inflammasome.^387^ Oridonin interacts with Cys279 of the NLRP3 NACHT domain via a covalent bond, inhibiting NLRP3-NEK7 interactions and attenuating the NLRP3 inflammasome activation.^388^ Tetrahydroquinoline, a synthesized compound, inhibits the NLRP3 inflammasome assembly and activation by binding to the NLRP3 NACHT domain and blocking ASC oligomerization.^389^ OLT1177, a β-sulfonyl nitrile compound, binds to NLRP3 directly to inhibit its ATPase activity, as well as to suppress caspase-1 activity and IL-1β production in monocytes from patients with cryopyrin-associated periodic syndrome.^390^ OLT1177 demonstrated excellent safety and tolerability in the phase I trial in heart failure and reduced ejection fraction in patients.^391^ β-Carotene binds to NLRP3’s PYD, suppressing NLRP3 inflammasome activation in macrophages induced by ATP, MSU crystals, and nigericin.^392^ 4-Methylenedioxy-β-nitrostyrene binds to NACHT and LRR domains of NLRP3 to inhibit its ATPase activity.^393^ Lycorine disrupts the interaction of NLRP3 with ASC by targeting the PYD on Leu9, Leu50, and Thr53.^394^ Anticancer agent tivantinib directly inhibits the NLRP3 ATPase activity and the subsequent assembly of the NLRP3 inflammasome complex.^395^ Bay 11-7082 was demonstrated to inhibit the ASC pyroptosome and the NLRP3 inflammasome organization via cysteine alkylation in the ATPase region of NLRP3.^396^ However, Bay 11-7082 also suppresses the IKKβ kinase activity, resulting in the modulation of NLRP3 expression via NF-κB pathway.^397^ 4-hydroxynonenal, a lipid peroxidation product, disrupts the interaction between NLRP3 and NEK7 via directly binding NLRP3.^398^ NIC-0102, an orally bioavailable proteasome inhibitor, promotes the NLRP3 polyubiquitination, disrupts the NLRP3-ASC interaction, and blocks ASC oligomerization, thus inhibiting activation of the NLRP3 inflammasome.^399^ INF39 suppresses the NEK7-NLRP3 interaction and, in turn, inhibits NLRP3-NLRP3 and NLRP3-ASC interactions, as well as ASC oligomerization and speckle formation.^400^

PYD-only protein POP3, an inhibitor of AIM2 inflammasomes, competes with ASC for AIM2 recruitment.^401^ Obovatol, a bisphenol chemical, inhibits the ASC pyroptosome formation and suppresses the NLRP3 and AIM2 inflammasome.^402^ RGFP966, a selective inhibitor of histone deacetylases 3, regulates the AIM2 spatiotemporally.^403^ DNA aptamers, small single-stranded DNA or RNA molecules, have the potential to bind to AIM2 and suppress its inflammasome activity.^404^ Roxadustat (FG-4592) inhibits AIM2 in a CD73-dependent manner.^405^

Recent research has found some products derived from medicinal plants or bioactive natural products can act as inhibitors of NLRP3 or AIM2 inflammasome. These will be valuable candidates for treating inflammasome-related diseases. Costunolide, the major active ingredient in the Chinese traditional medicinal herb Saussurea lappa, covalently binds to Cys598 in the NACHT domain of NLRP3 via the α-methylene-γ-butyrolactone motif in costunolide. This inhibits NLRP3 ATPase activity and NLRP3 inflammasome assembly.^406^ EFLA-945, an extract from red grapevine leaf, limits the DNA entry into THP-1-derived macrophages, thus inhibiting the AIM2 inflammasome activation.^407^ Ethanolic extracts derived from seeds of Cornus officinalis suppress AIM2 speck formation induced by dsDNA.^408^

### ASC modulation

As ASC is the adaptor protein of canonical inflammasomes, targeting ASC could also regulate inflammasome activation. J114, a chemical compound, disrupted interactions of NLRP3 or AIM2 with ASC and suppressed ASC oligomerization.^409^ It has been found that Caffeic acid phenethyl ester (CAPE) can directly bind to ASC, thereby preventing NLRP3-ASC interactions that are triggered by MSU crystals.^410^ A study involving a mouse model of gouty arthritis induced by MSU crystals showed that oral administration of CAPE resulted in a decrease in caspase-1 activation and IL-1β release in foot tissue and air pouch exudate. VHHASC, a type of alpaca single-domain antibody, impairs interactions between ASC and CARD by leaving the PYD of ASC functional, and by stabilizing an intermediate filament of inflammasome activation.^411^ Lonidamine, a small-molecule glycolysis inhibitor, directly binds to ASC and inhibits its oligomerization.^412^ Dehydrocostus lactone, a main component of traditional Chinese medicine Saussurea lappa, blocked the ASC oligomerization.^413^ Sulforaphane may directly disrupt the formation of NLRP3 via suppressing ASC or Caspase-1.^414^ Brevilin A, a natural ingredient derived from *Centipeda minima*, decreases the NLRP3 inflammasome activation by blocking the ASC oligomerization.^415^ 1,2,4-trimethoxybenzene, an active ingredient obtained from essential oils, inhibited ASC oligomerization as well as protein-protein interaction between NLRP3 and ASC.^416^

### Caspase modulation

Recently, the pharmaceutical industry has focused on developing inhibitors of caspase-1 protease, a component of all canonical inflammasomes. A substance called VX-765, also known as belnacasan, can help lessen the severity of AD by selectively inhibiting caspase-1.^417^ This is achieved through the covalent modification of catalytic cysteine residues. In addition, VX-765 has been found to improve cognitive impairments associated with AD and preserve ventricular functions, thereby ameliorating myocardial infarction in mice. Unfortunately, long-term exposure to VX-765 in animals has been shown to cause hepatotoxicity, preventing further development of the substance.^417^ Sennoside A, an ingredient in dietary supplements and weight-loss medicines, inhibits the enzymatic activity of caspase-1 to downregulate the NLRP3 and AIM2 inflammasome-involved inflammation dependent on P2X7.^418^ Pralnacasan, an orally absorbed nonpeptide compound, can inhibit caspase-1.^419^ Pralnacasan was shown to reduce joint damage in a collagenase-induced osteoarthritis mouse model, suggesting it could be used to treat osteoarthritis.^419^ Pralnacasan has also been shown to attenuate dextran sulfate sodium-induced murine colitis with little to no side effects.^420^

### IL-1/IL-18 modulators

IL-1β and IL-18 are the major inflammasome-activated inflammatory cytokines and play a significant role in the pathogenesis of several diseases. The biological anti-IL-1 agents have been approved for clinical application. Anakinra is a type of medication used to treat inflammatory diseases. It works as a recombinant interleukin-1 receptor antagonist.^421^ Patients with RA who are treated with anakinra experience a decrease in disease activity.^422^ A small randomized trial conducted at multiple centers found that anakinra significantly improved inflammatory and glycemic parameters in patients with both RA and T2D (NCT02236481).^423^ Canakinumab, which is a monoclonal antibody that targets human anti-IL-1β, has been approved by the US FDA and the European Medicines Agency as a treatment for cryopyrin-associated periodic syndromes.^424^ In a randomized, double-blind trial, canakinumab was found to be effective in reducing the recurrence rate of cardiovascular events compared to control group.^425^ Notably, anakinra requires more frequent injections and has a shorter half-life of only 4–6 h. Canakinumab, on the other hand, has a longer half-life of 26 days and has been shown in a randomized, double-blind clinical trial to provide better treatment response and increased safety for patients with active RA (NCT00784628). Rilonacept, an IL-1 inhibitor (IL-1 Trap) that is mainly used for the treatment of gout in children, was recently approved for a phase 3 trial in recurrent pericarditis.^426,427^ Gevokizumab, an IL-1β binding protein, possesses unique allosteric modulating properties.^428^ Patients with inflammatory and other diseases may benefit from gevokizumab.

IL-18 blockers are currently under investigation through clinical trials as well. One of these blockers is GSK1070806, which is a recombinant human IL-18 neutralizing antibody.^429^ A phase 1 clinical study was conducted on healthy and obese males with normal immune systems to determine the safety, efficacy, and antibody metabolism rate of GSK1070806. The study showed that GSK1070806 was generally well-tolerated, with positive results.^430^

### GSDMD modulators

GSDMD is the principal mediator of pyroptosis and directly triggers pyroptosis when inflammasomes are activated, making it a potential target for treating inflammasome-related diseases. Disulfiram is a drug that has been approved by the FDA for treating alcoholism. It works as an inhibitor of pore formation that is induced by GSDMD. This drug also blocks pore formation by covalently modifying Cys191/Cys192 in human/mouse GSDMD.^431^ When mice are treated with disulfiram, they show reduced pyroptosis, cytokine release, and LPS-induced septic death. It has been discovered that Necrosulfonamide can prevent the clustering of GSDMD by attaching to the Cys191 amino acid in GSDMD.^432^ This, in turn, reduces the incidence of neuronal pyroptosis triggered by Aβ in vivo. Additionally, Dimethyl fumarate can also impede GSDMD clustering and oligomerization.^433^ Further research is required to investigate the potential of GSDMD inhibitors for treating various ailments.

### Other modulators

In addition to the methods mentioned, other substances can affect inflammasomes by targeting the signaling pathways related to inflammasome activation. One of these substances, JC2-11, is a type of benzylideneacetophenone derivative that has shown promise as a potential pan-inflammasome inhibitor. Early research suggests that it can prevent caspase-1 cleavage, IL-1β release, GSDMD cleavage, and the production of mROS.^433^ Licochalcone B, a component of licorice, binds to NEK7 to suppress NLRP3 inflammasome activation by decreasing the interaction between NLRP3 and NEK7.^434^ Furthermore, pristimerin and ginsenoside Rg3 prevent the interaction between NEK7 and NLRP3, leading to reduced interaction between NLRP3 and ASC, decreased ASC oligomerization, and ultimately, reduced speckle formation.^435,436^ Methyl gallate inhibits the NLRP3 inflammasome assembly by blocking the ROS over-generation and NLRP3 oligomerization.^437^ 5-androstenediol promotes NF-κB signaling and suppresses inflammasome-mediated pyroptosis.^438^ Riboflavin downregulates mROS production and mDNA release, thus inhibiting the NLRP3 inflammasome assembly.^439^ Dihydroartemisinin induces autophagy to inhibit the AIM2 inflammasome and NF-κB/HIF-1α/VEGF pathways.^440^ ML365, the most potent TWIK2 channel blocker, inhibits ATP-induced NLRP3 inflammasome.^441^ Curcumin down-regulates NLRP1, caspase-1, GSDMD, and IL-1β in oxygen-glucose deprivation and middle cerebral artery occlusion models.^442^ Clinical trials found that N‐acetylcysteine, an antioxidant and anti‐inflammatory reagent, can reduce macrophagic expression of NLRP3, decrease IFN-γ production in NK cells, and subsequently reduce the synthesis and release of IL‐18.^443^ Glucocorticoids are widely used in treating COPD and AECOPD. A new anti-inflammatory drug, 17-oxo-DHA, when combined with hormones can inhibit the activation of the NLRP3 inflammasome and the release of mature IL-1β.^444^ Despite attempts to treat COPD patients with therapies targeting inflammasome-related effectors at moderate to severe stages, some randomized clinical trials have not shown significant benefits. For example, when COPD patients were infused with a human anti-IL-1β monoclonal antibody called canakinumab for 45 weeks, their forced expiratory volume in 1 s and forced vital capacity did not improve when compared to a placebo group. These results suggest that targeting inflammasome-related effectors may not have the desired effects. Therefore, large-scale, well-designed studies are necessary to draw a definitive conclusion. There are also some therapeutic candidates that are capable of inhibiting inflammasome activation but their mechanism is not currently understood. Fimasartan, phenolic and quinonemethide nor-triterpenes, dihydrotanshinone I, aryl sulfonamide derivatives, and tertiary sulfonylurea compounds could inhibt the NLRP3 or AIM2 inflammasome activation.^445–449^

## Conclusions

Inflammasomes play an irreplaceable role in multiple diseases. Understanding the mechanisms of activation and assembly of different inflammasomes and their functions in various conditions will provide valuable insights for effective intervention strategies in the future. This review introduced different inflammasomes, their structures, activation mechanisms, how they impact various diseases, and the potential therapeutic targets and intervention strategies. Inflammasomes are multiprotein complexes that play a vital role in regulating inflammatory and immune responses. Various types of inflammasomes have been identified, each characterized by different protein components. Understanding the distinct architectures of these inflammasomes is essential for elucidating their specific functions and developing targeted therapies for inflammatory diseases. Advanced structural studies on different inflammasomes have provided us with a brand-new perspective and approach to studying the mechanisms of inflammation activation. Inflammasome activation is associated with a conformation change of different sensor proteins, and the inflammasome assembly via homotypic interactions. Different inflammasomes are activated by specific stimuli, which trigger their assembly and the activation of caspase-1, leading to the maturation of proinflammatory cytokines like IL-1β and IL-18. The canonical pathway triggers inflammasome activation in response to a two-step process. The first step involves the activation of PRRs which detect PAMPs or DAMPs and activate NF-κB to induce transcription of NLRP3 and proinflammatory cytokines. Once NLRP3 has been upregulated, the second step involves the assembly of the inflammasome complex and subsequent caspase-1 activation. The non-canonical pathway of inflammasome activation is triggered by the activation of caspase-4 or -5 (caspase-11 in mice) in response to cytosolic LPS from Gram-negative bacteria. Caspase-4/5 cleaves GSDMD, leading to the release of proinflammatory cytokines and pyroptosis. Alternative pathways include the activation of inflammasomes by other PRRs like RIG-I-like receptors (RLRs) or DNA sensors like AIM2. These pathways involve different inflammasome components and mechanisms for their activation. Understanding the different inflammasome activation pathways helps develop potential therapies for multiple diseases. New research has made important strides in comprehending how certain inflammasomes contribute to various human ailments. This knowledge can be useful in discovering fresh treatments for metabolic, neurodegenerative, and inflammatory conditions. It is essential to identify the roles of specific inflammasomes in systemic diseases, so that targeted therapeutic approaches can be developed to regulate their activities and reduce inflammation. The identification of therapeutic targets and the creation of inflammasome-targeted strategies have yielded encouraging results in both lab and clinical trials.

There are still many unanswered questions in the research field of inflammasomes. The activation of inflammasomes has different pathways, including canonical and non-canonical, with multiple processes involved. The intricate process of inflammasome activation also involves multiple signaling pathways. Understanding these mechanisms fully remains a challenge. Although there are some inhibitors available, the development of highly selective and potent inhibitors targeting specific inflammasome components is still in progress. Additionally, the regulatory mechanisms controlling inflammasome activity and its interplay with other immune pathways, such as autophagy and cytokine signaling, are not fully understood. Animal models used to study inflammasome-related diseases may not fully replicate human physiology, making it difficult to apply findings from animal models to human clinical settings. Moreover, it can be difficult to determine the specific contributions of different inflammasomes in disease states due to their overlapping functions. While inflammasomes have been linked to various diseases, the use of inflammasome-targeted therapies in clinical practice is currently limited. Further research is required to bridge this gap and enhance our knowledge of inflammasome biology. This could lead to the development of safe and effective treatments that modulate inflammasome activity in disease settings. More investigations are necessary to fully understand the role of inflammasomes in disease pathogenesis and to address any controversies. As research in this area continues, we can expect to see better clinical outcomes and a greater understanding of the relationship between inflammation and human diseases.
